# Mitoxantrone and abacavir: An ALK protein-targeted *in silico* proposal for the treatment of non-small cell lung cancer

**DOI:** 10.1371/journal.pone.0295966

**Published:** 2024-02-06

**Authors:** Juan Enrique Faya Castillo, Richard Junior Zapata Dongo, Paolo Alberto Wong Chero, Stefany Fiorella Infante Varillas

**Affiliations:** 1 Departamento de Ciencias Básicas, Bioética y la Vida Humana, Facultad de Medicina Humana, Universidad de Piura, Lima, Perú; 2 Departamento de Ciencias de la Medicina, Facultad de Medicina Humana, Universidad de Piura, Lima, Perú; Saveetha University - Poonamallee Campus: SIMATS Deemed University, INDIA

## Abstract

Non-small cell lung cancer (NSCLC) is a type of lung cancer associated with translocation of the EML4 and ALK genes on the short arm of chromosome 2. This leads to the development of an aberrant protein kinase with a deregulated catalytic domain, the cdALK^+^. Currently, different ALK inhibitors (iALKs) have been proposed to treat ALK^+^ NSCLC patients. However, the recent resistance to iALKs stimulates the exploration of new iALKs for NSCLC. Here, we describe an *in silico* approach to finding FDA-approved drugs that can be used by pharmacological repositioning as iALK. We used homology modelling to obtain a structural model of cdALK^+^ protein and then performed molecular docking and molecular dynamics of the complex cdALK^+^-iALKs to generate the pharmacophore model. The pharmacophore was used to identify potential iALKs from FDA-approved drugs library by ligand-based virtual screening. Four pharmacophores with different atomistic characteristics were generated, resulting in six drugs that satisfied the proposed atomistic positions and coupled at the ATP-binding site. Mitoxantrone, riboflavin and abacavir exhibit the best interaction energies with 228.29, 165.40 and 133.48 KJoul/mol respectively. In addition, the special literature proposed these drugs for other types of diseases due to pharmacological repositioning. This study proposes FDA-approved drugs with ALK inhibitory characteristics. Moreover, we identified pharmacophores sites that can be tested with other pharmacological libraries.

## 1. Introduction

Lung cancer (LC) is one of the major causes of cancer death worldwide and the most frequent cancer in men [1]. Although in the last decades there has been considerable research to understand its biological and clinical aspects, the overall survival remains poor [1]. Lung cancer is a very heterogeneous group and different parameters are used to classify them. From a histological point of view there are two types: small cell lung cancer (SCLC) and non-small cell lung cancer (NSCLC) [2, 3].

Molecular and clinical studies of NSCLC have identified functional mutations in tyrosine kinase (TK) receptors such as anaplastic lymphoma kinase (ALK), present in ~7% of adenocarcinoma tumors that can be used in targeted therapy [4]. Targeted therapy for ALK has shown better results in progression-free survival (PFS) and quality of life in patients with ALK^+^ NSCLC, compared to chemotherapy [5]. Thus, understanding the ALK genomic architecture, alterations and physicochemical activities could help us understand further candidates to be implemented in the clinical practice.

Hence, ALK gene, located in 2p23, was initially described in a non-Hodgkin´s lymphoma [6], associated with typical chromosomal translocation t(2;5)(p23;q35) [7]. ALK alterations are present in NSCLC as a chromosomal rearrangement involving paracentric inversion inv(2)(p21p23) with echinoderm microtubule-associated protein-like 4 (EML4) gene located in 2p21. EML4-ALK fusion gene exhibits different variants (V) caused by variability of EML4 breakpoints onto exon 20 of the ALK gene, there are eight variants of EML4-ALK (from V1 to V8), where V1 and V3 a/b are the most recurrent (75–80%) [8, 9]. This genomic rearrangement generates an aberrant protein with tumorigenic activity, where the ALK protein loses its extracellular and transmembrane domain, while TK domain remains in the cytoplasmic region. The TK domain is autophosphorylated and activated by the trimerisation domain, which is part of EML4 N-terminal domain [10–12]. The catalytic domain of EML4-ALK (cdALK^+^) has two lobes (N-terminal and C-terminal) hinged through a short linker region, harbouring a catalytic cleft in the middle of the lobes. Moreover, ALK uses ATP as phosphate group donator for ALK substrates phosphorylation. The cdALK^+^ targeted therapy drugs compete with ATP-binding site located in N-terminal lobe [13]. (ALK structure is shown in S1 Fig).

Currently, there are available some cdALK^+^ inhibitors (iALK^+^) for the treatment of ALK^+^ NSCLC patients: crizotinib as first generation; ceritinib, alectinib; brigatinib, lorlatinib and ensartinib from second and third generation, respectively [14–16]. Despite the efficacy of iALK^+^ in patients with ALK^+^ NSCLC, therapeutic resistance has been detected [17].

The resistance mechanisms are depending and non-depending on ALK [18, 19]. Resistance to crizotinib mainly occurs near the cdALK^+^, close to ATP-binding site. This change reduces the crizotinib interaction thus activating signalling pathways of cell proliferation and survival [20]. This acquired resistance bear secondary cdALK^+^ mutations such as L1196M and S1206Y, L1152P/R and C1156Y/T, and G1202R, in crizotinib-resistant, crizotinib and ceritinib, and crizotinib-ceritinib-alectinib resistant tumours, respectively [11]. These secondary mutations lead to structural changes that avoid iALK^+^ interactions with ATP-binding site, but not affecting the cdALK^+^-ATP interaction [21].

Resistance to iALK^+^ has motivated the search for new drugs or molecules for ALK^+^ NSCLC treatment. Due to high costs of experiments studies, computational approaches seem to be of help for a rapid determination of iALK^+^ in ALK^+^ NSCLC. One of the techniques used is the drug repositioning (DR), to find new therapeutic uses of existing drugs. Therefore, our objective was to computationally evaluate drugs and/or molecules that may play a possible role in inhibiting cdALK^+^-mediated phosphorylation in ALK^+^ NSCLC. The proposed drugs have been approved by institutions such as FDA or EMA, moreover their medical prescription is not originally proposed for NSCLC or is still under investigation with clinical trials [22, 23].

## 2. Materials and methods

### 2.1 Preparations for pharmacophoric model

Preparing the pharmacophoric model is a critical step in the search for drugs that can fulfill a drug repositioning role, for that reason, the following steps were performed prior to pharmacophore-based virtual screening.

#### 2.1.1 Molecular modeling of cdALK^+^

The cdALK^+^ sequence (UniProt code: J7MA22) was used for homology modelling with YASARA 21.6.2 (http://www.yasara.org/)., which presents the optimized functions so that the low-energy protein has a better chance of being modeled correctly in order to achieve local minima that is closer to native structure. YASARA also generates a hybrid using the best parts of the generated models to increase the accuracy of the model, in case the models based on the template are not optimal [24].

#### 2.1.2 Molecular docking of cdALK^+^ with ALK^+^ inhibitors

Before the process / analysis / evaluation of molecular dynamics of the complex cdALK^+^-iALK^+^, the cdALK^+^ co-crystallized with their inhibitors crizotinib; ceritinib, alectinib, brigatinib; and lorlatinib (PDB [25] code: 5AAA, 4MKC, 3AOX, 6MX8 and 5AA9, respectively) were structurally aligned with our cdALK^+^ model. The structures of cdALK^+^ crystallized were removed leaving only the co-crystallized inhibitors, now in complex with modeled cdALK^+^. The new complex was cleaned, and the missing hydrogens were added, finally an energy minimization was performed on YASARA™ [26]. The binding energy between the ligand and the receptor was calculated in addition to being saved with a “.sce” extension to proceed with the molecular dynamics. The molecular docking technique in drug repositioning is widely used to deduce the relationships that exist between the substrate-proteins, allowing to predict the binding of the ligands of interest at the desired binding site as explained in the research of Abdou A. *et al*. [27–30].

#### 2.1.3 Molecular dynamics of cdALK^+^ with FDA-approved drugs

For this step, the YASARA™ software was used to perform the simulations of the cdALK^+^ with ATP and its known inhibitors: crizotinib, ceritinib, brigatinib, alectinib, lorlatinib. The AMBER 14 force fields [31] was chosen for the molecular dynamics and a periodic cubic box was generated around the complex with an extension of 20 Å with physiological pH configurations at 7.4 and 0.9% NaCl for ion concentration as a mass fraction. The system simulation created was neutralized with intermolecular potential3 points (TIP3P) water molecules as solvent with a density of 0.997 g/l, (moreover) the temperature used was 298 K and a pressure of 1 atm, coulomb electrostatics at a cutoff of 8Å (the default used by AMBER). The simulation time step was 2 ft and each trajectory was saved at 100 ps. The simulation was carried out for 150 ns, to later analyze using the YASARA™ macro(md_analyze_dynamics.mcr), the root mean square deviation (RMSD), root mean square fluctuation (RMSF), radius of gyration (Rg) and interaction bonds to obtain the key amino acids throughout the molecular dynamics to generate the pharmacophore [32, 33].

### 2.2 Pharmacophore generation and virtual screening

The pharmacophoric model was performed on Pharmit [34] web server, based on the cdALK^+^-ATP and cdALK^+^-inhibitor complexes. Four pharmacophores were created and saved in “.json” format to be used for virtual screening from the contributed library called FDA-aproved drugs, containing 21582 conformers of 1856 molecules. The size was restricted to the ATP interaction pocket, considering the most relevant selected molecular characteristics that include the spatial arrangement of these characteristics, a tolerance of 1 was used in the "Exclusive shape" option. The resulting drugs were then coupled with cdALK^+^ to determine their binding energy. The generated model was subjected to molecular dynamics with the same characteristics mentioned above and analyzed with md_analyze_dynamics.mcr.

## 3. Results

### 3.1 Pharmacophore model generation

#### 3.1.1 Modeling and molecular docking of cdALK^+^ with iALK exhibit better binding energy than ATP

**Fig 1:**
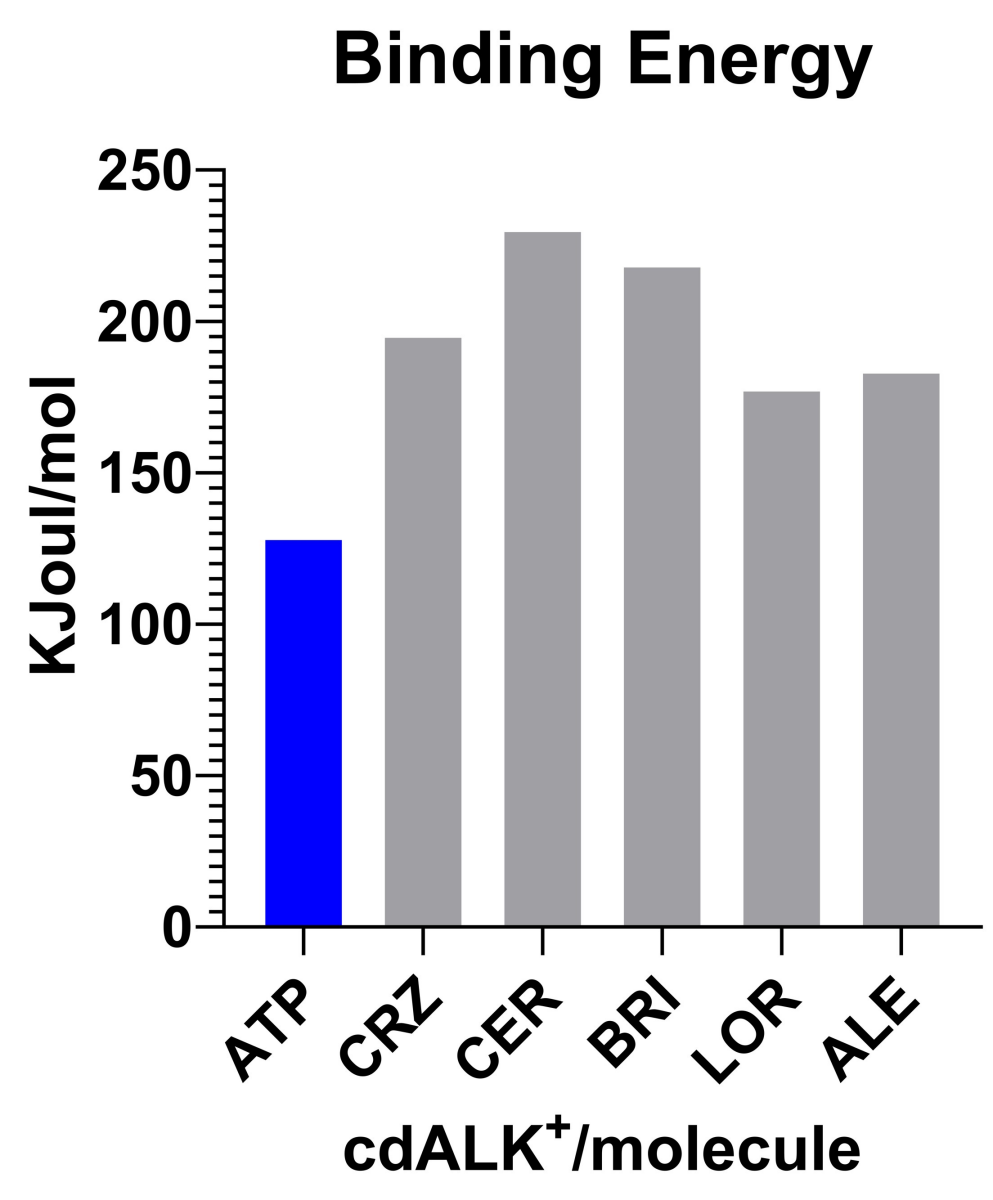
Bar graphs of the binding energy in *KJoul/mol* of each iALK drug compared to the ATP molecule, interacting with cdALK^+^. It can be seen that the iALKs have higher interaction energies. CRZ (crizotinib), CER (ceritinib), ALE (alectinib), BRI (brigatinib) and LOR (lorlatinib).

The structure of cdALK^+^ was modelled with YASARA™. Different models were generated using the three-dimensional structures as templates (PDB code: 4CLJ, 4FOD, 4ANL, 5FTO) stored in RCSB-PDB (https://www.rcsb.org/) obtaining a hybrid model that combines the best parts of the models previously obtained with each template, thus increasing the precision of the final model; these PDB templates were chosen since they present the structure of cdALK^+^ in complex with the specific inhibitors, but the structure is not complete. The Z-score of the hybrid model was 0.115 (Overall = 0.145*Dihedrals + 0.390*Packing1D + 0.465*Packing3D). Since the structure of cdALK^+^ in complex with ATP has not been resolved experimentally, we used cdALK in complex with ADP (PDB code: 3LCT) to couple the different ligands into our model. To model cdALK^+^-ATP complex structure, ADP molecules from cdALK were removed and then ATP from Human cyclin-dependent kinase 2 (PDB code: 1B39) was docked to obtain a new complex, which was later was minimized with YASARA™. The binding energy between ATP and cdALK^+^ in this complex was of 128.33 KJoul/mol. The binding energy between iALK with cdALK^+^ was measured with the same strategy. The interaction of cdALK^+^ with ceritinib obtained the highest value (230.04 KJoul/mol), followed by brigatinib (218.34 KJoul/mol), crizotinib (195.12 KJoul/mol), alectinib (183.34 KJoul/mol) and lorlatinib (177 KJoul/mol). All iALKs (S2 Fig) presented values higher than ATP as shown in Fig 1 and S1 Table in S1 File.

#### 3.1.2 Molecular dynamics of cdALK^+^ with iALK

**Fig 2:**
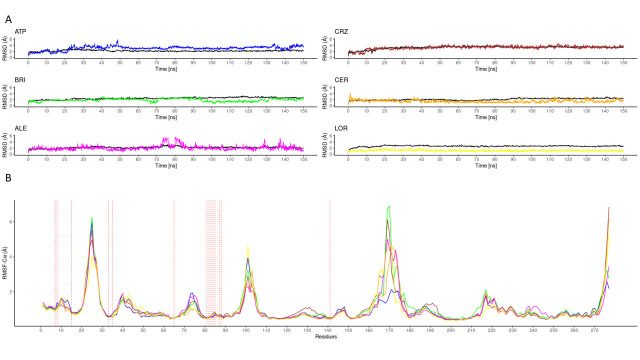
RMSD values of the molecular dynamics. A) RMSD values of iALK in colors (ATP: blue, crizotinib: ccep, brigatinib: green, ceritinib: orange, alectinib: magenta and lorlatinib: yellow), in black RMSD-Cα values of cdALK^+^. B) RMSF-Cα values of cdALK^+^, the amino acid that interacting with the iALK are shown with perpendicular lines to x axis.

**Fig 3:**
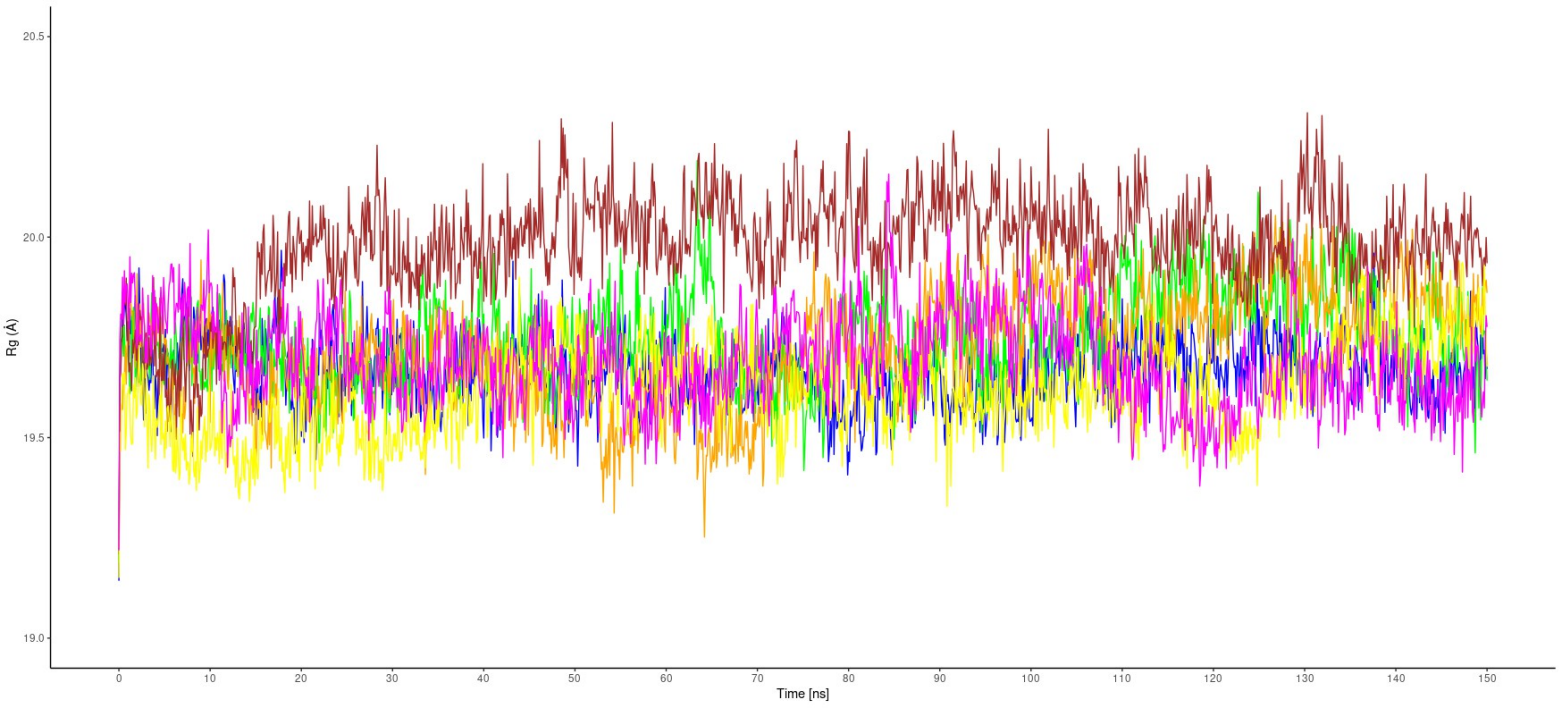
Radius of gyrations (Rg) values of iALK drugs and ATP molecule with cdALK^+^. ATP: blue, crizotinib: brown, brigatinib: green, ceritinib: orange, alectinib: magenta and lorlatinib: yellow.

The cdALK^+^-iALK complexes were then analyzed by molecular dynamics over 150 ns. The root-mean-square deviation (RMSD) of alpha carbon (Cα) value of cdALK^+^-ATP and cdALK^+^-iALK was compared with each other to determine the structure stabilities throughout molecular dynamics (Fig 2A). RMSD-Cα of cdALK^+^-ATP (2.18 ± 0.22; mean ± standard deviation) was similar to cdALK^+^-iALK (2.52 ± 0.33). In addition, the radius of gyrations value of cdALK^+^-ATP and cdALK^+^-iALK were 19.67 ± 0.08 and 19.75 ± 0.12, respectively, confirming the minimal structural changes between the compared models (Fig 3 and S3 Fig).

The RMSD value of its ATP molecule and iALK drugs were also measured throughout the molecular dynamics (Fig 2A). Ceritinib, alectinib and lorlatinib had the lowest RMSD values: 1.5 Å, 2.1 Å and 1.2 Å respectively, compared to 3.01 Å of ATP. The RMSD of crizotinib and brigatinib was very similar to ATP molecule with 3.2 Å and 3.6 Å respectively.

The molecular dynamics of the cdALK^+^-iALKs complexes during the 150 ns provided insight into the molecular interactions between them. The amino acids shown in S4 Fig exhibit the interaction with both hydrogen bonds and/or hydrophobic interactions throughout the molecular dynamics. Met82, Met84 and Ala85 were the amino acids with the highest frequency of hydrogen bonds with iALKs (S2 Table in S1 File). The amino acids Leu7, Gly8, Val15, Ala33, Tyr35, Val65, Leu81, Leu83, Gly87, Leu88, Leu141, presented proportions of hydrophobic interactions in more than 50% of the molecular dynamics (S2 Table in S1 File). To reinforce this result, the values of root-mean-square fluctuation (RMSF)-Cα of the protein complexes were analyzed. The amino acids previously mentioned had less than 2 Å of RMSF-Cα in all molecular dynamics (Fig 2B). Moreover, iALK drugs and ATP molecule had similar values of RMSF-Cα. Finally, all these results were used to generate the pharmacophore.

#### 3.1.3 Pharmacophore model generation

The results obtained with PLIP^TM^ from average structure of cdALK^+^-iALKs reveal that the amino acids that present hydrogen bonds are Glu82 (crizotinib and lorlatinib), Met84 (all iALKs) and Leu-88 (lorlatinib) (S4A Fig). The hydrophobic interactions between cdALK^+^-iALK shown in S4B Fig are also present in PLIP™ results.

The pharmacophore generated with information collected from molecular dynamics, interaction profiles and specialized literature was made with at least 5 defined characteristics shown in Table 1. The characteristics proposed were: two donor atoms that generate hydrogen bonds with the backbone of the amino acids Glu82 and Met84 (HBDo1 and HBDo2) from the interactions of all iALK; one receptor atom that generates hydrogen bonds with the backbone of the amino acid Met84 (HBA) from the interactions of all iALK; two aromatic elements that represent the rings (Aro1, Aro2) found in positions of hydrophobic elements (Hyd 1 and Hyd2); and one extra aromatic element in the pocket between β4 –β5 and αC-glutamate to prevent interaction between Lys35 and Glu52 (Aro3, prerequisite for the activated state of ALK).

**Table 1:** Receptor-ligand based pharmacophore models and their distinct characteristics.

Model	Characteristics
Pharmacophore 1 (S5 Fig)	HBDo1, HBDo2, HBA, Hyd 1, Hyd 2, Aro1
Pharmacophore 2 (S6 Fig)	HBDo1, HBDo2, HBA, Hyd 1, Hyd 2
Pharmacophore 3 (S7 Fig)	HBDo1, HBDo2, HBA, Hyd 2, Aro 1
Pharmacophore 4 (S8 Fig)	HBDo1, HBDo2, HBA, Aro 2, Aro 3

HBDo: hydrogen bond donor, HBA: hydrogen bond aceptor, Hyd: hydrophobic, Aro: aromatic.

### 3.2 Virtual screening

The generated pharmacophores were used as a query to search for drugs that meet the selected characteristics in the virtual library of FDA-approved drugs available at PHARMIT™. The results of the pharmacophoric models are detailed in Table 2 and the atoms positions are representing in S5–S8 Figs:

- Pharmacophore 1 and 2: Uracil mustard, inamrinone and minoxidil.
- Pharmacophore 3: Inamrinone, abacavir and uracil mustard.
- Pharmacophore 4: Riboflavin and mitoxantrone.

**Table 2:** Result of virtual screening based on the pharmacophoric model.

Drugs	RMSD (Å)	Binding Energy (KJoul/mol)	Hydrogen Bond	Hydrophobic interactions	Pi-stacking
**Riboflavin**	0.724	165.40	Glu52 Glu82 Met84 Phe156	Leu141	Lys35
**Inamrinone**	0.746	87.67	Glu82 Met84	Val15 Leu141	
**Abacavir**	0.762	133.48	Glu82 Met84 Asn139	Val15 Leu141	
**Uracil mustard**	0.763	88.89	Glu82 Met84		
**Mitoxantrone**	0.808	228.29	Ala11 Lys35 Glu82 Met84 Arg138 Asp15	Val15	
**Minoxidil**	0.870	96.48	Glu82 Met84	Leu141	

**Fig 4:**
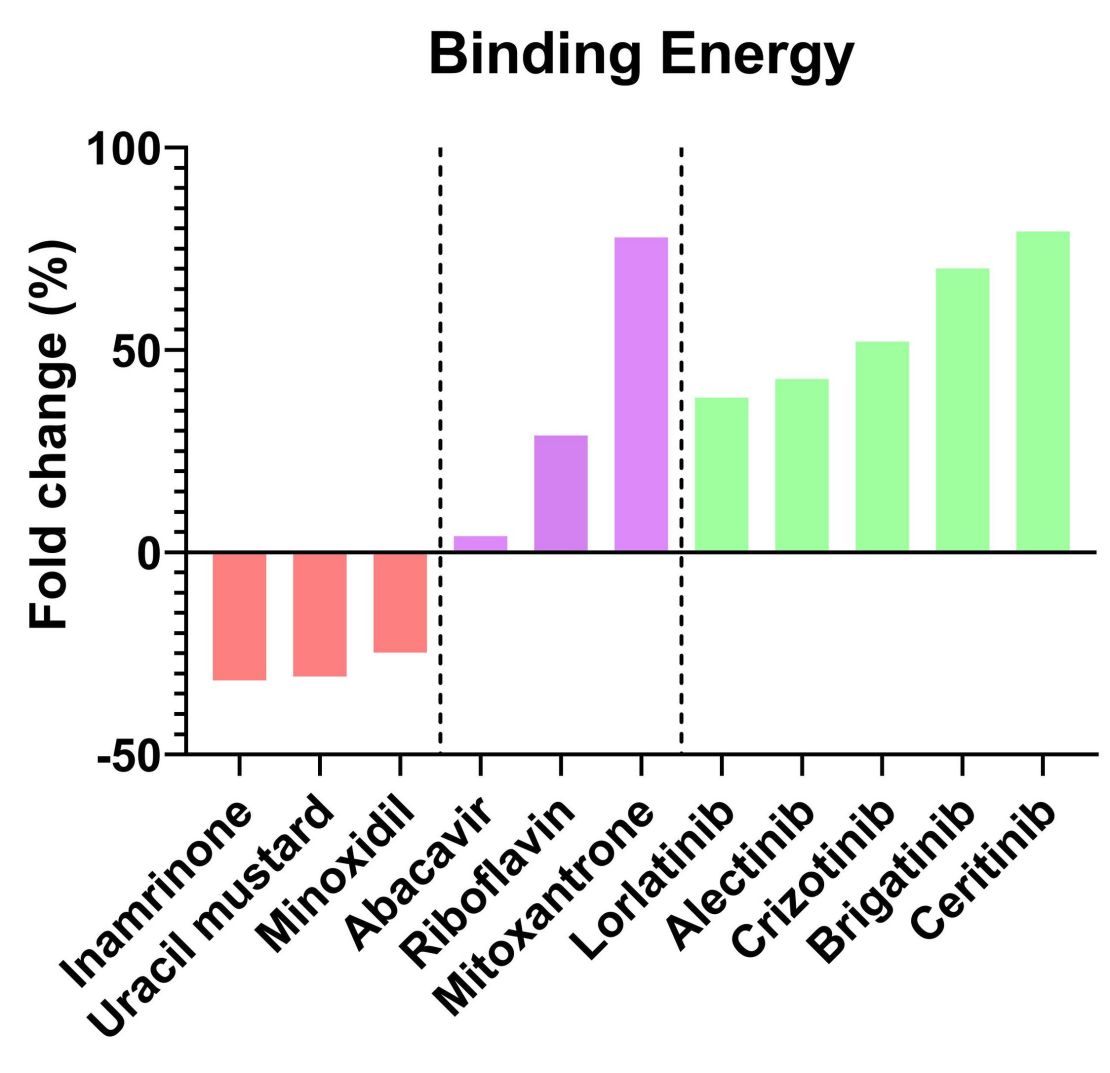
Fold change of binding energy between drugs and cdALK^+^. Comparison of fold change in percentage (%) of the binding energy between cdALK^+^ -iALK and cdALK^+^-FDA-approved drugs. Red color: Molecules exhibit lower binding energy than ATP (Inamrinone, Uracil mustard, Minoxidil); Magenta color: Molecules exhibit major binding energy than ATP (Abacavir, Riboflavin and Mitoxantrone); Green color: iALK exhibit major binding energy than ATP (Lorlatinib, Alectinib, Crizotinib, Brigatinib and Ceritinib).

**Fig 5:**
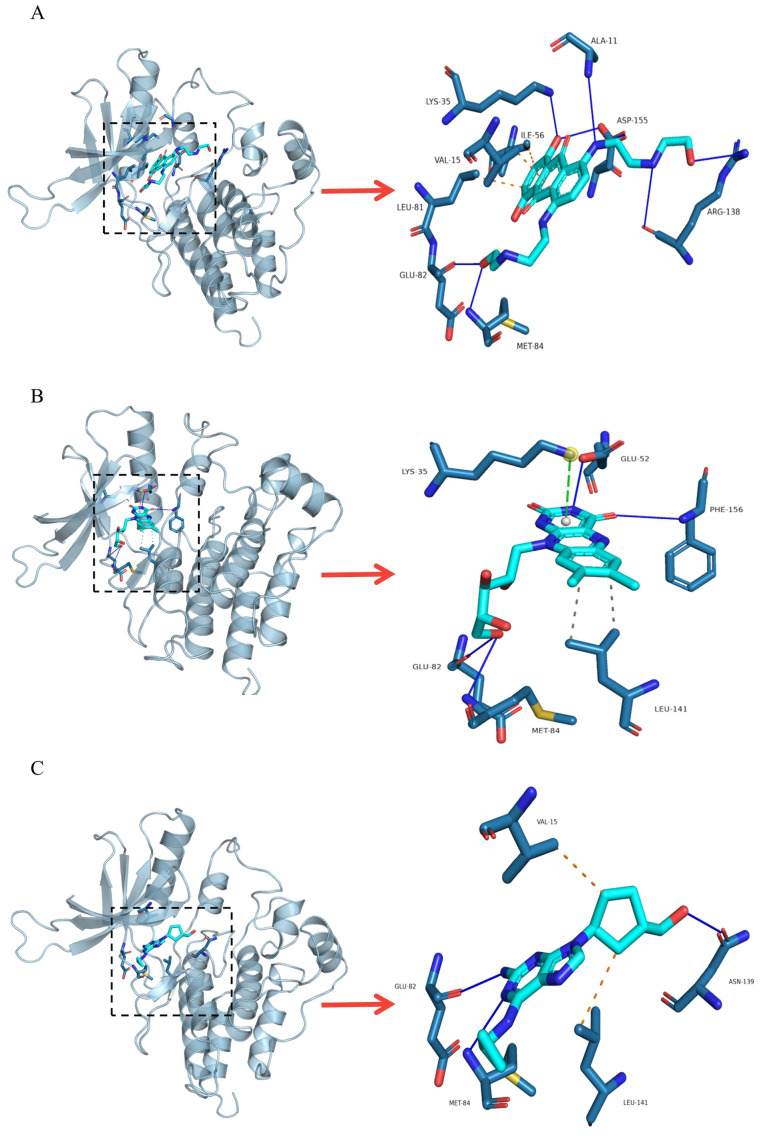
Interactions between cdALK^+^/ FDA-approved drugs. A) cd ALK^+^/mitoxantrone, B) cdALK^+^/riboflavin, C) cdALK^+^/abacavir.

FDA-approved drugs obtained in the virtual screening had RMSD values below 1 Å and hydrogen bonds with at least the two most important amino acids Glu82 and Met84. The selected drugs were six and the results reveal that mitoxantrone, abacavir and riboflavin present a higher binding energy than found in the cdALK^+^-ATP complex (128.33 KJoul/mol). Besides, molecular docking reveals that mitoxantrone has a higher binding energy than the molecular coupling of the reference iALK drugs., only below ceritinib (230.04 KJoul/mol) (Fig 4). Furthermore, these drugs interact with the same amino acids previously found in iALK drugs with molecular dynamics (S4 Fig). The interactions of the protein complexes of cdALK^+^-mitoxantrone, cdALK^+^-riboflavin, cdALK^+^-abacavir are represented (Fig 5), showing the arrangement of the amino acids that interact with the iALKs mentioned in the Table 2.

### 3.3 Molecular dynamics of complexes cdALK^+^/mitoxantrone, cdALK^+^/riboflavin and cdALK^+^/abacavir

**Fig 6:**
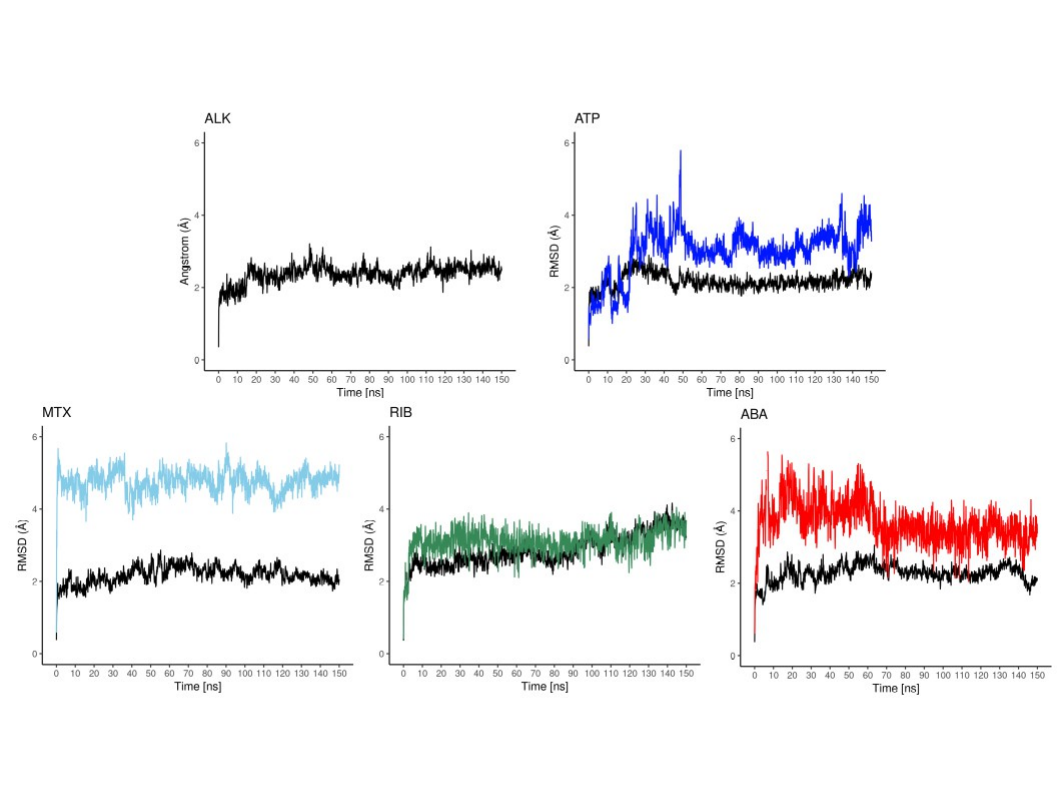
RMSD LigMove values of ATP: Blue, mitoxantrone (MTX): Blue sky, riboflavin (RIB): Dark green, abacavir (ABA): Red, in black RMSD-Cα values of cdALK^+^.

**Fig 7:**
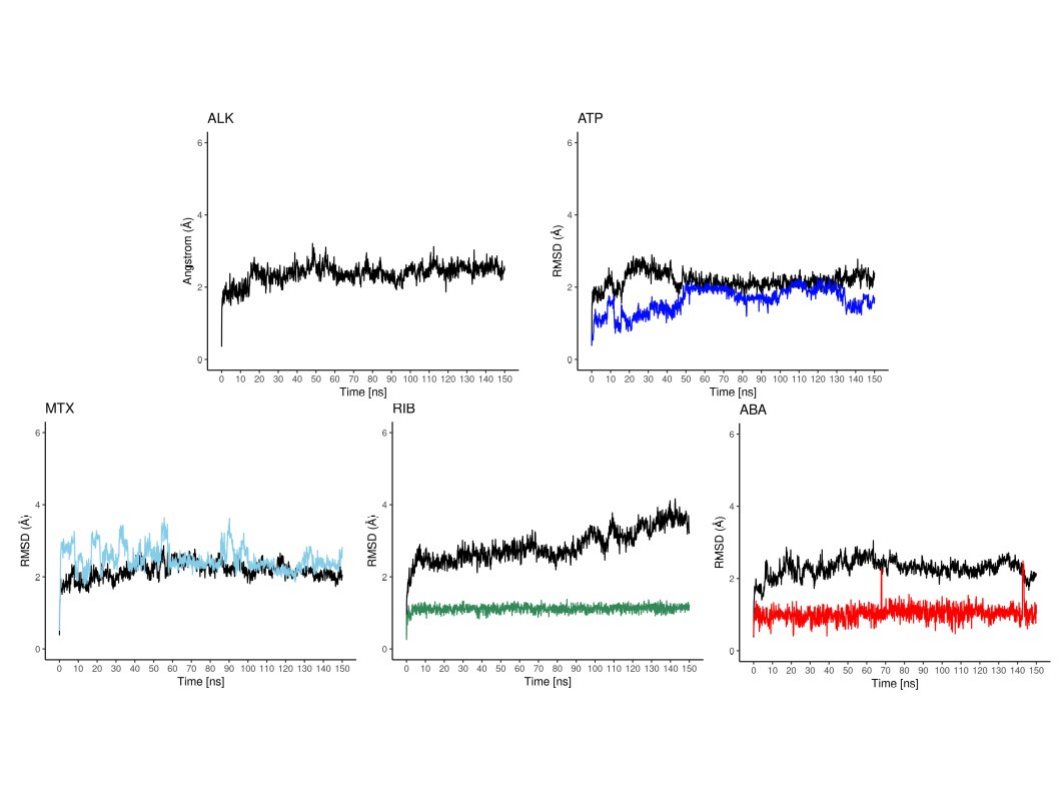
RMSD LigConf values of ATP: Blue, mitoxantrone (MTX): Blue sky, riboflavin (RIB): Dark green, abacavir (ABA): Red, in black RMSD-Cα values of cdALK^+^.

The analysis of the molecular dynamics of the selected drugs, reveals that cdALK^+^-mitoxantrone complex had an RMSD-Cα of 2.17 ± 0.24, cdALK^+^-riboflavin complex had an RMSD-Cα of 2.86 ± 0.45 and cdALK^+^-abacavir complex had an RMSD-Cα of 2.27 ± 0.25. Relative to the drugs, the RMSD LigMove (Fig 6) of mitoxantrone, riboflavin and abacavir, were 4.75 ± 0.34, 3.11 ± 0.36 and 3.68 ± 0.54, respectively. RMSD LigMove delivers information about the movement of the ligand in its binding pocket. The RMSD LigConf (Fig 7) of the drugs were 2.51 ± 0.33, 1.12 ± 0.95 and 1.03 ± 0.21 to mitoxantrone, riboflavin and abacavir, respectively. RMSD LigConf summarize the conformational changes of the ligand. The results of radius of gyration were obtained to and the comparation with iALKs are details in the S3 Table (S1 File).

**Fig 8:**
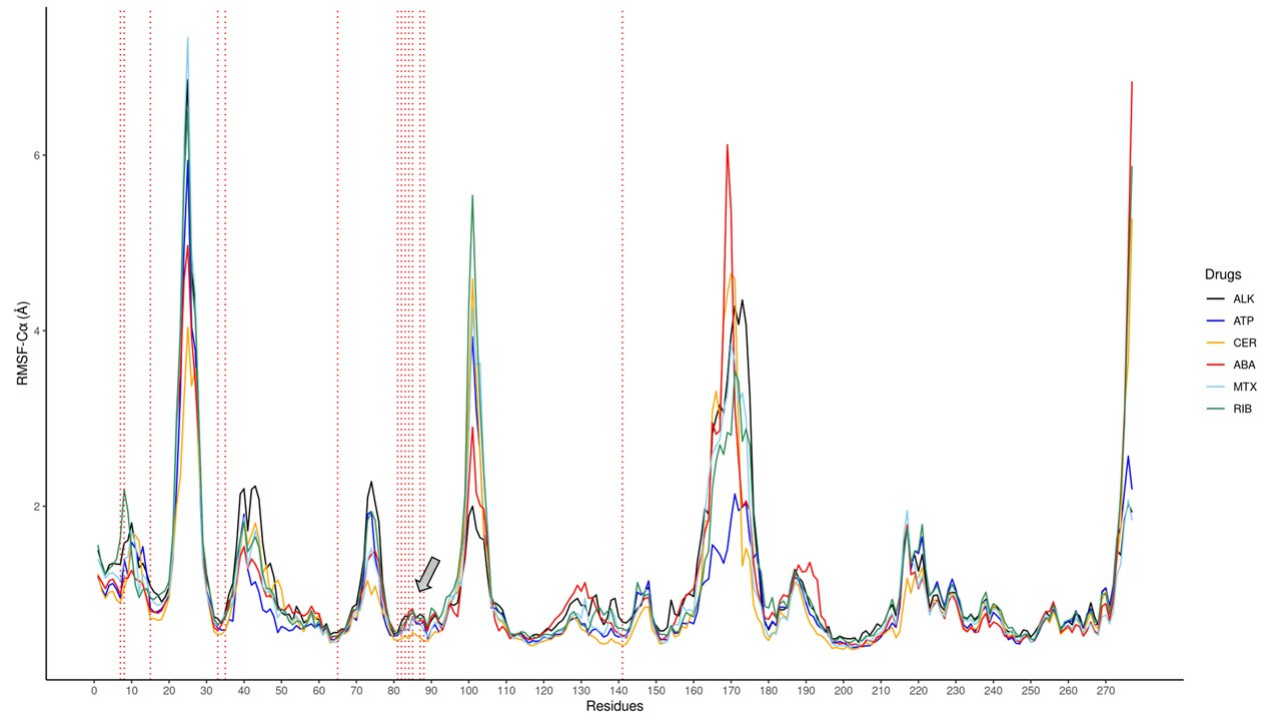
RMSF-Cα values of cdALK^+^ in interactions with abacavir (ABA, red), mitoxantrone (MTX, blue sky), riboflavin (RIB, dark green), certinib (CER, orange), ATP (blue). cdALK^+^ without inhibitor is in black line. The important aminoacids that interact with cdALK^+^ are found in perpendicular lines to the x-axis. P-loop is pointed with an arrow.

In relation to RMSF, the RMSF-Cα of cdALK^+^ protein in its apo conformation versus RMSF-Cα cdALK^+^ interacting with mitoxantrone, riboflavin, abacavir and certinib were compared, the latter having the best interaction energy according to the results mentioned in Fig 1. The results show that the amino acids that normally interact with iALKs behave similarly in abacavir, riboflavin and mitoxantrone. The stability of the loop between the amino acids Leu80 –Asp88 where the key aminoacids of interaction with iALKs are found, has also been compromised. In Fig 8 the amino acids of the P-loop (pointed with an arrow) are more stable in the ALK^+^/abacavir complex than in its apo form or with the ALK^+^/ceritinib and ALK^+^/ATP complexes.

In the Fig 8, the regulatory αC-chain comprised between amino acids Val40 –Asn60 has a behavior similar to those observed in the ALK^+^/ceritinib and ALK^+^/ATP complexes, with a tendency to decrease in movement as they approach amino acid Val65. Since ALK is a protein kinase, it presents a catalytic triad formed by the amino acids Asp155, Phe156 and Gly157; necessary for the phosphorylation process. Preceding the catalytic triad, there is an activation segment between Arg160 and Trp180, which is more stable in the ALK^+^/ATP complex (blue line) unlike the other complexes, this may be due to the fact that there must be a suitable environment for the donation of the phosphate group of ATP to the substrate. In the case of cdALK^+^ complexes with iALK^+^, mitoxantrone and abacavir the activation segment is less stable and thus phosphorylation would not be allowed.

## 4. Discussion

Despite the existence of iALKs for ALK^+^ NSCLC patients, resistance to iALKs has been seen across disease progression. Resistance to crizotinib, ceritinib, brigatinib, alectinib, and lorlatinib is produced by specific mutations in the ATP-binding site [35]. Therefore, understanding the cdALK^+^-iALK interaction allows us to propose molecules and/or drugs with potential pharmacological repositioning capacity. In our study, we use computational techniques (molecular modelling, molecular docking, molecular dynamics, and virtual screening) to identify new characteristics in existing drugs to propose them as candidates for pharmacological repositioning for ALK^+^ NSCLC treatment.

Molecular docking and subsequent analysis in PLIP™ showed that iALK and ATP interact through hydrogen bonds with the carboxyl group of Glu82 and the amino group of Met84. Throughout the dynamics, these aminoacids appear interacting with iALK and ATP more than 50% of times, revealing their importance. Glu82 and Met84 are part of the ALK hinge region together with Leu83, Ala85 and Gly86. According with Zhang et al. [36], the majority of small-molecule inhibitors of protein kinases that compete with ATP interact with the backbone of residues of hinge region. Our study suggests that the drugs found with potential pharmacological repositioning interact with the same amino acids (Table 2 and S4 Fig).

Mitoxantrone is a drug that inhibits DNA replication and transcription by intercalating DNA. In our study using PLIP^TM^, MTX interacts through seven hydrogen bonds with cdALK^+^, with the amino acids Ala11, Lys35, Ile56, Glu82, Met84, Arg138, and Asp155. It also performs three hydrophobic interactions with the amino acids Val15, Ile56 and Leu81. Ile56 is in the regulatory helix αC, which is part of a buried pocket, in this domain, the distal part of the planar dihydroxyanthraquinone of MTX lodges generating a hydrophobic interaction between the side chain of Ile56 and the H19 atom of MTX. In addition, Glu52 of the regulatory αC-helix could generate a hydrogen bond between the OE2 atom of the amino side chain with the O4 atom of MTX. On the other hand, extensive charged and polar interactions are formed between the flexible hydroxyethylamino residues of MTX with the amino acids of cdALK^+^.

All these interactions explain the greater binding energy value of MTX (228.20 Kjoul /mol) compared to the other proposed drugs, including iALKs (except for ceritinib [230.04 Kjoul /mol]). These findings reveal that MTX would be a good ATP-competitive ALK inhibitor. Studies about MTX have demonstrated therapeutic efficacy in advanced breast cancer, non-Hodgkin’s lymphoma, acute non-lymphoblastic leukaemia, and chronic myelogenous leukemia [37, 38]. *In vitro* trials have also demonstrated an effect of MTX against formation of lung metastases, related to inhibition of their vascularization [39]. In addition, MTX would have a role as a potent inhibitor of ROS1 demonstrated by *in silico* and *in vitro* studies in NSCLC cells. This activity relays in its ability to suppress ROS1 phosphorylation and, subsequently, inducing cell apoptosis [40]. However, there are also few reports on the effect of MXT on NSCLC lines at the cellular level.

Studies have shown that MTX can interact with protein kinases, Xiaobo Wan and co-workers in 2013, conducted a study where they computationally coupled MTX with 143 kinases in active conformation, the best 5 results were tested in vitro, and PIM1 kinase validated MTX to be used as an inhibitor in cancer cells because MTX showed to possess low nanomolar inhibitory activity against PIM1 kinase and to inhibit the PIM1-mediated phosphorylation in cancer cells [41]. In the same year, Golubovskaya, proposed MTX as an antineoplastic agent by interacting with the kinase domain of Focal Adhesion Kinase (FAK), a non-receptor kinase that is overexpressed in many types of tumors [42]. In the same way Guan in 2020, by means of experimental tests, indicated that MTX could bind with Eukaryotic elongation factor 2 kinase and inhibit its activity [43]. Thus, MTX appears to be a good candidate as a cdALK^+^ inhibitor that should be further studied in preclinical and clinical settings.

Riboflavin belongs to the vitamin B family (B2 vitamin), that exhibits antioxidant, anti-inflammatory, antinociceptive and antitumoral properties [44, 45]. Previous studies have already reported that dietary B vitamin (RIB, among them) may reduce breast cancer risk [46]. Other studies have demonstrated that irradiated RIB on human renal carcinoma cell line exhibit antitumor activity by inducing intrinsic apoptosis pathway [47]. *In vitro* and *in vivo* assays in melanoma also evidenced that irradiated RIB treatment may disrupt cell migration [48]. In addition, RIB combined with cisplatin (CP) as adjuvant could reduce the CP-induced toxicity and induce apoptosis in mouse skin cancer model [49]. It is important to mention that RIB has been tested in LC models and it shows that high doses of RIB in ALK^+^NSCLC models could promote tumorigenic progression [50] but, RIB has not been tested for ALK^+^ NSCLC, the antitumoral role of RIB and its interaction with the cdALK^+^ (with similar mechanisms of FDA-approved iALK such as CRZ, ALE, BRI, CER, and LOR) reinforces the need for further experiments assessing its effect as iALK. Our results suggest that RIB fits into EML4-ALK protein as a potent ATP-competitive molecule due to higher binding energy metrics than ATP and, although it is lower than other FDA-approved iALK, it seems attractive as a potential pharmacological subject of study for ALK^+^ NSCLC patients.

Abacavir is a reverse transcriptase inhibitor used against human immunodeficiency virus type 1 (HIV-1). In our study, abacavir interacts with cdALK^+^ within three hydrogens bonds: Glu82, Met84 and Asn139, and it has two hydrophobic interactions with Val15 and Leu141, generating a binding energy value of 133.48 Kjoul /mol. Glu82 and Met84 bind the Abacavir rings at the ATP binding site by hydrogen bond interaction, the cyclo-propane moiety does not exhibit interactions, being free throughout the dynamics. The hydroxymethyl cyclopentane portion exhibits hydrogen bond interactions between the O atom of Abacavir and the OD1 atom of the Asn139 side chain. LigPlot+^TM^ analyses shows the O atom of the carboxyl group of Arg138 as a hydrogen bond with the O atom of Abacavir. Although Abacavir does not occupy the buried portion of cdALK^+^ throughout the dynamics, it can still be considered as a candidate for cdALK+ inhibition. RMSD and RMSF analysis demonstrate Abacavir behaves like iALKs, which could be tested in further *in vitro* experiments. In other types of cancers, studies exploring abacavir observed the reduction of cell growth, migration and invasion processes in prostate cancer cell lines [51]. In breast cancer cells, abacavir had good results by increasing apoptosis [52]. Moreover, *in silico* and *in vitro* studies conducted by Beklen et al. proposed abacavir for colorectal cancer for its statistically significant inhibition profiles on the CRC cell line [53].

Finally, in the literature review conducted by Doumat and co-workers in 2023, they present a section called “Anti-Retroviral Drugs” where they propose different anti-retroviral drugs for the treatment of HIV approved for NSCLC repositioning, examples such as lopinavir, ritonavir, efavirenz and nelfinavir are exposed [54]. In this way, we propose the anti-retroviral abacavir as a potential repositioned drug for different types of cancer including NSCLC.

## 5. Conclusions

The results obtained by virtual screening of defined pharmacophores reveal six drugs with atomistic positions that complies the proposed characteristics given by the iALKS analysis in complex with cdALK^+^. Three of them (ribloflavin, abacavir and mitoxantrone), exhibit high affinity with the interaction pocket of ATP-binding site like iALKs in the same pocket. This lets us to consider these drugs as important candidates for pharmacological reposition. The interaction mechanisms of these drugs are based on hydrogen bonds with Glu82 and Met84 amino acids, which belong to hinge residues of interaction for iALKs. Our results reveal that the three drugs under study present similar coupling to the iALKs clinically used (crizotinib, ceritinib, brigatinib, alectinib and lorlatinib) showing encouraging results that can be tested in vitro. Although riboflavin exhibits superior binding energy to ATP, previous studies demonstrated its adverse activity by increasing cell proliferation and migration in A549 and H3255 non-small cell lung cancer cell models. Hence, riboflavin does not appear to be a promising candidate for the suggested pharmacological repositioning. However, the structural attributes shared between Riboflavin and the other two drugs offer a valuable foundation for the development of novel ATP-competitive anti-tumoral for ALK^+^NSCLC. Nevertheless, with results obtained, we propose two new drugs, mitoxantrone and abacavir as more suitable candidates to assessment as iALK by its higher affinity metrics to the cdALK^+^ structure.

## Supporting information

S1 File Supplementary tables. (DOCX)

S2 File Abbreviations. (DOCX)

S1 Fig Catalityc domain of human ALK. Ribbon diagram of catalytic domain of human ALK. The cdALK^+^ has two lobes, the cyan small N-terminal and orange large C-terminal. The N-terminal lobe contain five-stranded antiparallel β-sheet and regulatory αC-helix (in magenta), between β-1 and β-2 exist a conserved glycine-rich with ATP-phosphate-binding loop (in red) called P-loop. P-loop is followed by a conserved valine (in green and stick format). The C-terminal has an activation region with DFG amino acid residues (in blue), in sticks format the activation segment tyrosine phosphorylation sites (in orange). In yellow the key amino acids in the interaction with ALK inhibitors.
(TIF)

S2 Fig FDA-approved ALK inhibitors. Structure of the iALKs in riboon format.
(TIF)

S3 Fig A) Cartoon representations of the cdALK^+^. Amino acids with more than 50% interactions with iALK are coloured in red, whereas the molecular surface of ATP-binding site is shown in green. B) Lines of representation of ATP-binding site amino acids, in yellow the key amino acids in the interaction with ALK inhibitors with its respective names and positions.
(TIF)

S4 Fig Heatmap of aminaocid that interact with cdALK^+^/iALK and cdALK^+^/FDA-approved drugs. A) shows the aminoacids that have hydrogen bond interaction with the drugs under study, in addition to the rate throughout all the dynamics. B) shows the amino acids that have hydrophobic bond interactions with the drugs under study, in addition to the rate throughout all the dynamics. The yellow squares are amino acid that are not present in the interactions with iALK or FDA-approved drugs.
(ZIP)

S5 Fig Pharmacophore 1. HBDo: hydrogen bond donor, HBA: hydrogen bond aceptor, Hyd: hydrophobic, Aro: aromatic.
(TIF)

S6 Fig Pharmacophore 2. HBDo: hydrogen bond donor, HBA: hydrogen bond aceptor, Hyd: hydrophobic, Aro: aromatic.
(TIF)

S7 Fig Pharmacophore 3. HBDo: hydrogen bond donor, HBA: hydrogen bond aceptor, Hyd: hydrophobic, Aro: aromatic.
(TIF)

S8 Fig Pharmacophore 4. HBDo: hydrogen bond donor, HBA: hydrogen bond aceptor, Hyd: hydrophobic, Aro: aromatic.
(TIF)
