# Supplementary material for: Mitoxantrone and abacavir: An ALK protein-targeted in silico proposal for the treatment of non-small cell lung cancer
Source: PLoS One. 2024 Feb 6;19(2):e0295966. doi: 10.1371/journal.pone.0295966 (PMC10846704; doi:10.1371/journal.pone.0295966)
Supplement: S1 File — (DOCX) [file pone.0295966.s001.docx]

**Supplementary tables**

**S1 Table: Binding energy between cdALK^+^ and iALK**

| Protein complex | Binding energy |
| --- | --- |
| cdALK^+^-ATP | 128.33 KJoul/mol |
| cdALK^+^-crizotinib | 195.12 KJoul/mol |
| cdALK^+^-ceritinib | 230.04 KJoul/mol |
| cdALK^+^-alectinib | 183.34 KJoul/mol |
| cdALK^+^-brigatinib | 218.34 KJoul/mol |
| cdALK^+^-lorlatinib | 177.38 KJoul/mol |

This table shows the binding energy between ATP and iALK with cdALK^+^. The binding energy values of iALK are higher than those of ATP.

**S2 Table: Hydrogen bond between cdALK^+^/iALK with its respective porcents.**

| Hydrogens bonds | | | | | | | | | |
| --- | --- | --- | --- | --- | --- | --- | --- | --- | --- |
| CRZ | | **BRI** | | **CER** | | **ALE** | | **LOR** | |
| AA | **%** | **AA** | **%** | **AA** | **%** | **AA** | **%** | **AA** | **%** |
| Glu82 | 93% | Met84 | 80% | Met84 | 63% | Met84 | 75% | Glu82 | 98% |
| Ala85 | 60% |  |  |  |  |  |  |  |  |

This table shows the percentage of the most recurrent amino acids along the molecular dynamics that interact with hydrogen bonds with cdALK**^+^**.

**S3 Table:** **Comparations of the RMSD between iALKs and proposed drugs**

| RMSD | | | | |
| --- | --- | --- | --- | --- |
| **Complex** | **RMSD Cα** | **LigMove** | **LigConf** | **Rg** |
| cdALK^+^-ATP | 2.17 ± 0.23 | 3.01 ± 0.67 | 1.64 ± 0.35 | 19.67 ± 0.08 |
| cdALK^+^-CRZ | 3.22 ± 0.48 | 3.23 ± 0.60 | 1.82 ± 0.35 | 19.98 ± 0.13 |
| cdALK^+^-BRI | 2.45 ± 0.26 | 3.55 ± 1.11 | 1.90 ± 0.46 | 19.75 ± 0.11 |
| cdALK^+^-CER | 2.12 ± 0.32 | 1.51 ± 0.43 | 1.02 ± 0.25 | 19.72 ± 0.14 |
| cdALK^+^-ALE | 2.18 ± 0.29 | 2.07 ± 0.79 | 1.09 ± 0.39 | 19.69 ± 0.12 |
| cdALK^+^-LOR | 2.62 ± 0.23 | 1.21 ± 0.23 | 0.65 ± 0.13 | 19.61 ± 0.11 |
| cdALK^+^-MTX | 2.17 ± 0.24 | 4.75 ± 0.34 | 2.51 ± 0.33 | 19.68 ± 0.11 |
| cdALK^+^-RIB | 2.86 ± 0.45 | 3.11 ± 0.36 | 1.12 ± 0.95 | 20.05 ± 0.19 |
| cdALK^+^-ABA | 2.27 ± 0.25 | 3.68 ± 0.54 | 1.03 ± 0.21 | 19.73 ± 0.11 |

RMSD-Cα compares the behavior of cdALK^+^ protein in interaction with iALKs and proposed drug. LigMove is referenced to ligand heavy atoms over time, this procedure delivers information about the movement of the ligand in its binding pocket. LigConf is referenced ligand atoms over time, the gained data summarize the conformational changes of the ligand. (Yasara^TM^). The Radius of gyrations (Rg) delivers information about the compactness of a protein its relationship with the folding. All values are represented by their mean ± standard deviation.
