# Supplementary material for: Mitoxantrone and abacavir: An ALK protein-targeted in silico proposal for the treatment of non-small cell lung cancer
Source: PLoS One. 2024 Feb 6;19(2):e0295966. doi: 10.1371/journal.pone.0295966 (PMC10846704; doi:10.1371/journal.pone.0295966)
Supplement: S2 File — (DOCX) [file pone.0295966.s002.docx]

**Abbreviations**

ABA: Abacavir

ADP: Adenosine diphosphate

ALE: Alectinib

ALK: Anaplastic lymphoma kinase

ALK^+^: Abnormal ALK protein

ALK^+^ NSCLC: NSCLC caused by abnormal ALK protein

AMBER: Assisted Model Building with Energy Refinement

Aro: Aromatic

ATP: Adenosine triphosphate

BRI: Brigatinib

Cα: Alpha carbon

CER: Ceritinib

cdALK: The catalytic domain of ALK

cdALK^+^: The catalytic domain of EML4-ALK

CP: Cisplatin

CRZ: Crizotinib

DR: Drug repositioning

EMA: European Medicines Agency

EML4: Echinoderm microtubule-associated protein-like 4

FDA: Food and Drug Administrations

HBA: Hydrogen bond aceptor

HBDo: Hydrogen bond donor

Hyd: Hydrophobic

iALK^+^: cdALK^+^ inhibitors

LC: Lung cancer

LOR: Lorlatinib

PDB: Protein Data Bank

PFS: Progression free survival

PLIP: Protein-Ligand Interaction Profiler

MTX: Mitoxantrone

NSCLC: Non-small cell lung cancer

RCSB: Research Collaboratory for Structural Bioinformatics

RIB: Riboflavin

Rg: Radius of gyration

RMSD: Root mean square deviation

RMSF: Root mean square fluctuation

SCLC: Small cell lung cancer

TK: Tyrosine kinase

UniProt: Universal Protein Data Base

V: Variants

YASARA: Yet Another Scientific Artificial Reality Application
