# Supplementary figures and images for: Mitoxantrone and abacavir: An ALK protein-targeted in silico proposal for the treatment of non-small cell lung cancer
Source: PLoS One. 2024 Feb 6;19(2):e0295966. doi: 10.1371/journal.pone.0295966 (PMC10846704; doi:10.1371/journal.pone.0295966)

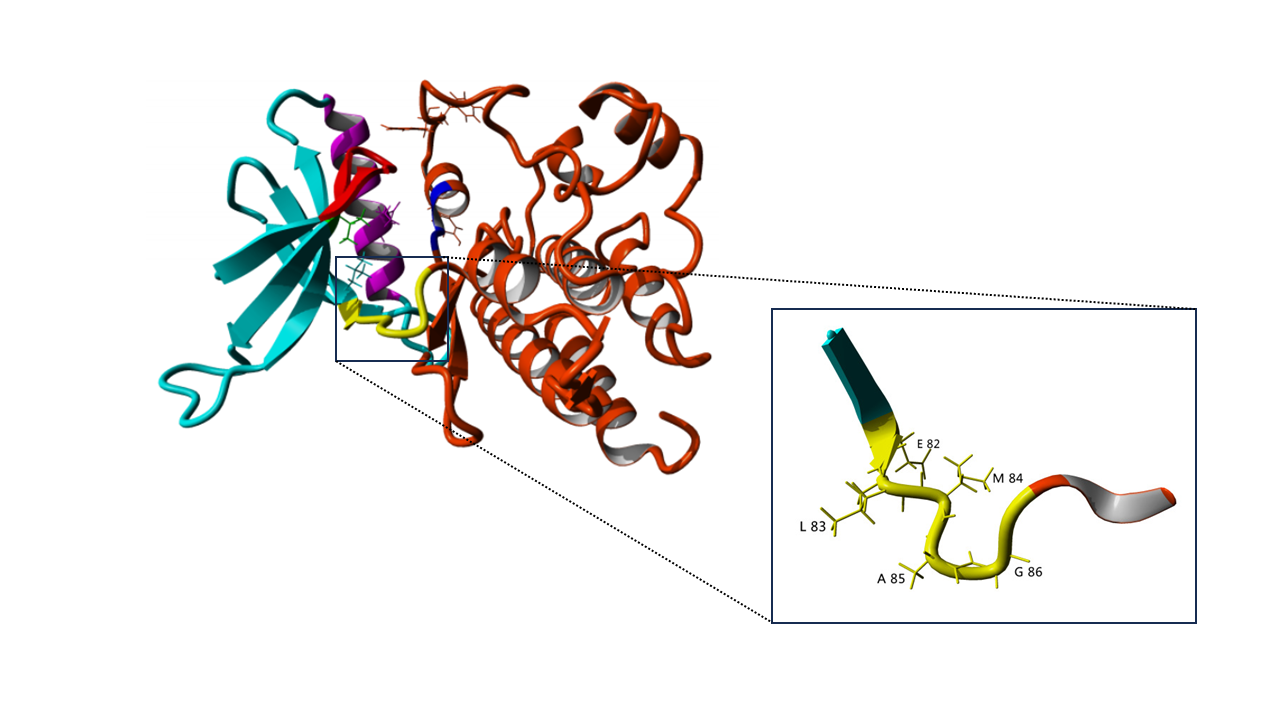

Supplement: S1 Fig — Ribbon diagram of catalytic domain of human ALK. The cdALK+ has two lobes, the cyan small N-terminal and orange large C-terminal. The N-terminal lobe contain five-stranded antiparallel β-sheet and regulatory αC-helix (in magenta), between β-1 and β-2 exist a conserved glycine-rich with ATP-phosphate-binding loop (in red) called P-loop. P-loop is followed by a conserved valine (in green and stick format). The C-terminal has an activation region with DFG amino acid residues (in blue), in sticks format the activation segment tyrosine phosphorylation sites (in orange). In yellow the key amino acids in the interaction with ALK inhibitors. (TIF) [file pone.0295966.s003.tif]

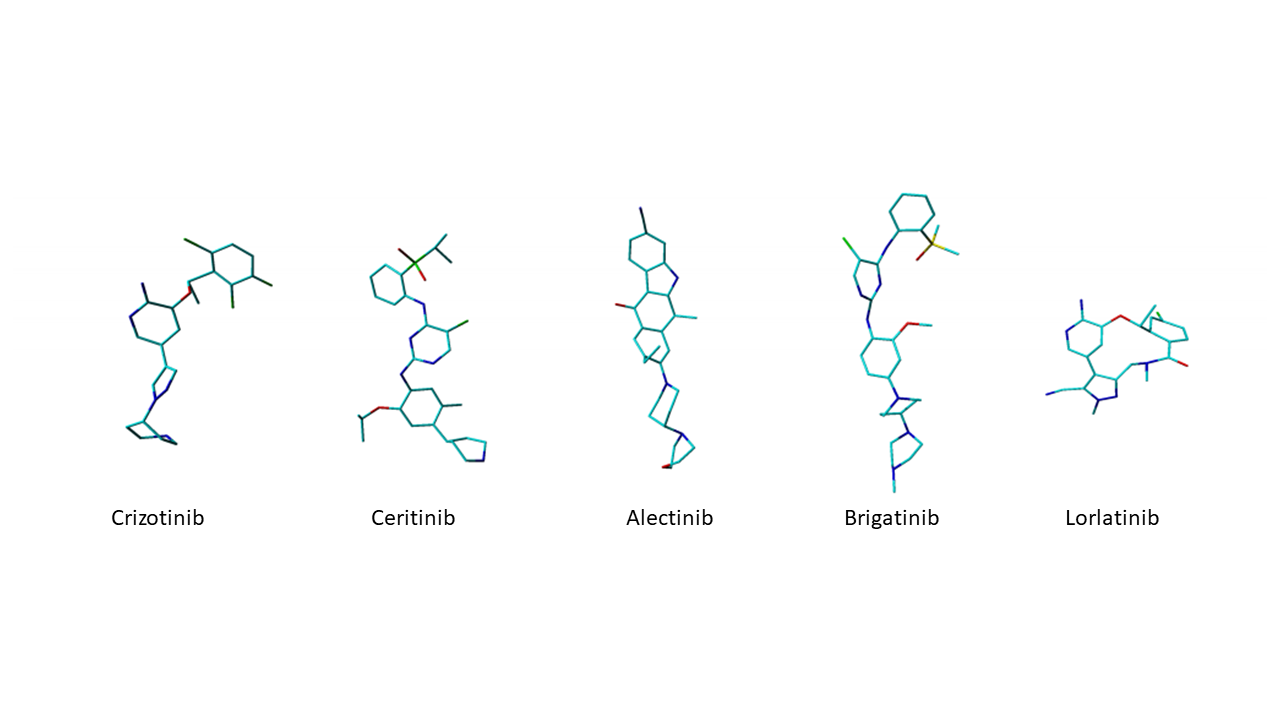

Supplement: S2 Fig — Structure of the iALKs in riboon format. (TIF) [file pone.0295966.s004.tif]

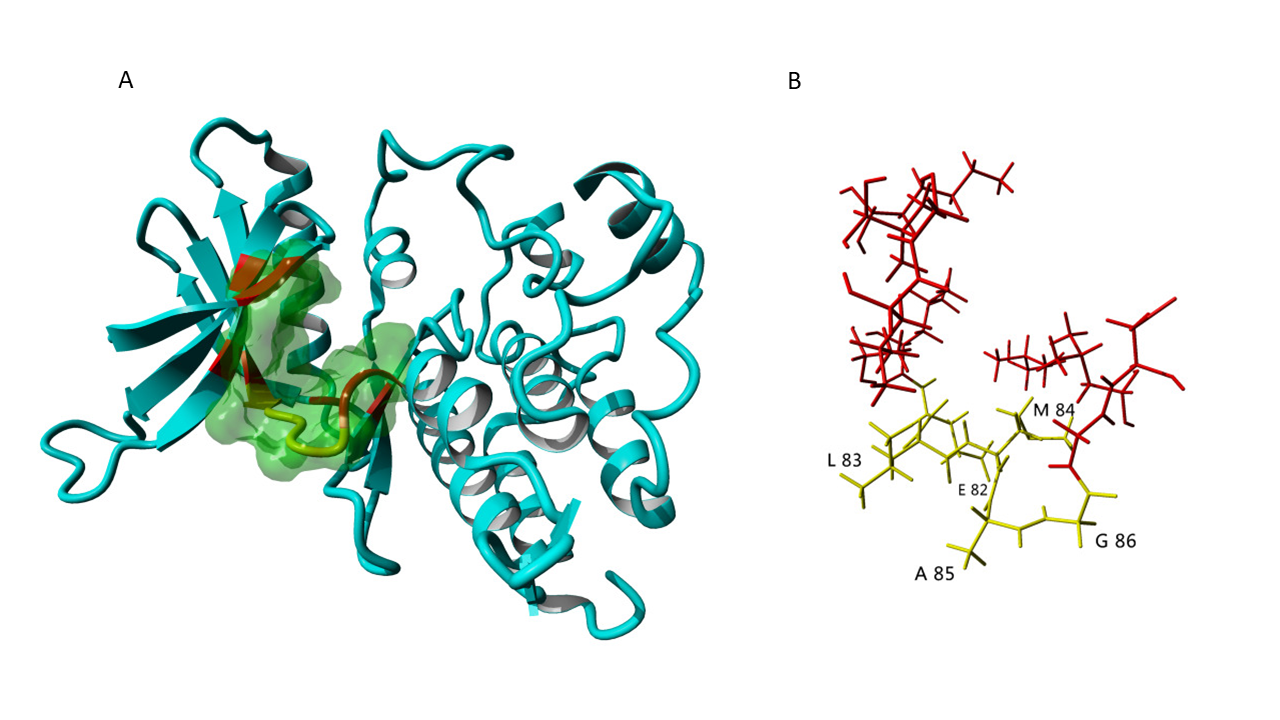

Supplement: S3 Fig — A) Cartoon representations of the cdALK+. Amino acids with more than 50% interactions with iALK are coloured in red, whereas the molecular surface of ATP-binding site is shown in green. B) Lines of representation of ATP-binding site amino acids, in yellow the key amino acids in the interaction with ALK inhibitors with its respective names and positions. (TIF) [file pone.0295966.s005.tif]

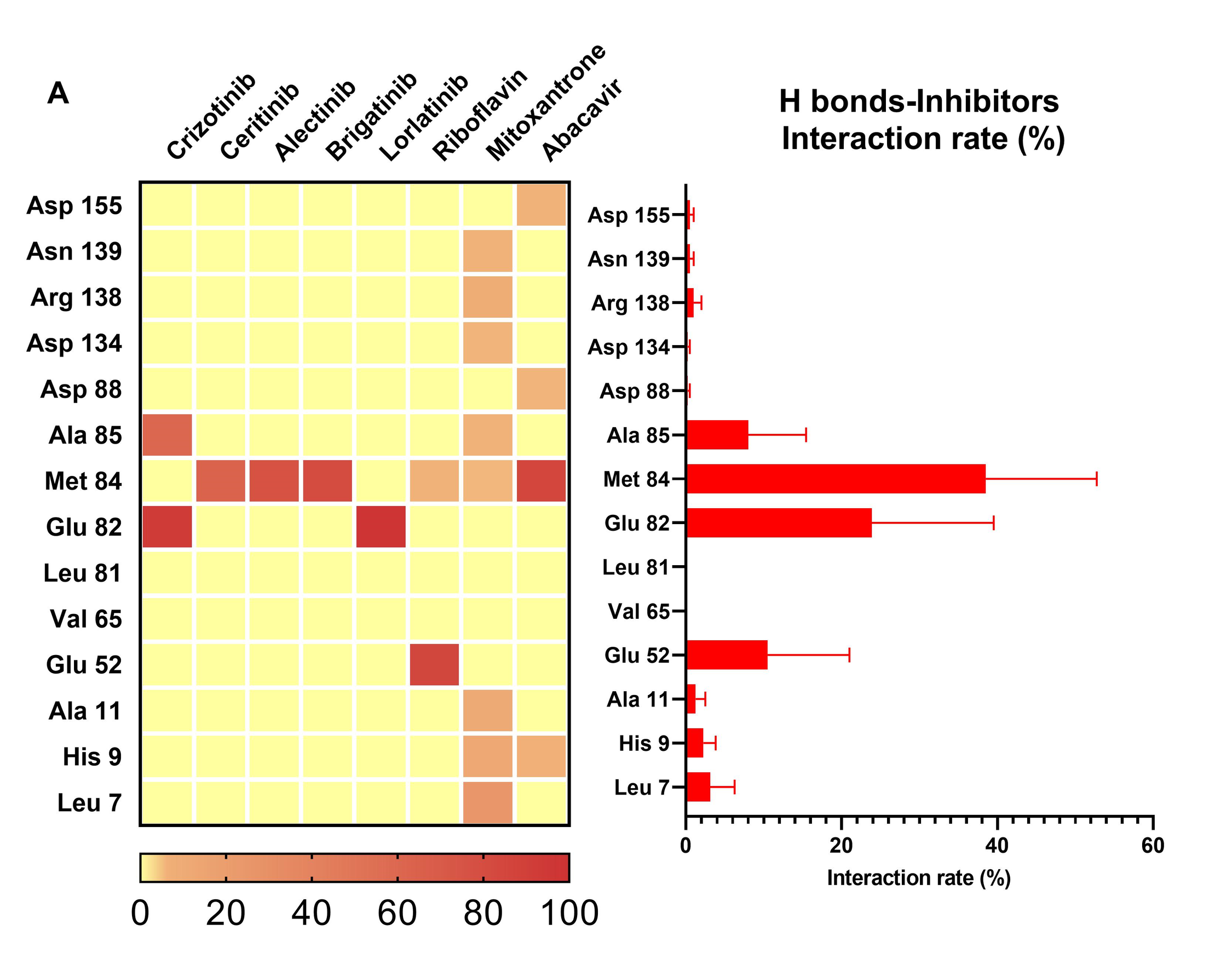

Supplement: S4 Fig — A) shows the aminoacids that have hydrogen bond interaction with the drugs under study, in addition to the rate throughout all the dynamics. B) shows the amino acids that have hydrophobic bond interactions with the drugs under study, in addition to the rate throughout all the dynamics. The yellow squares are amino acid that are not present in the interactions with iALK or FDA-approved drugs. (ZIP) [file pone.0295966.s006.zip › S4A_Fig.tif]

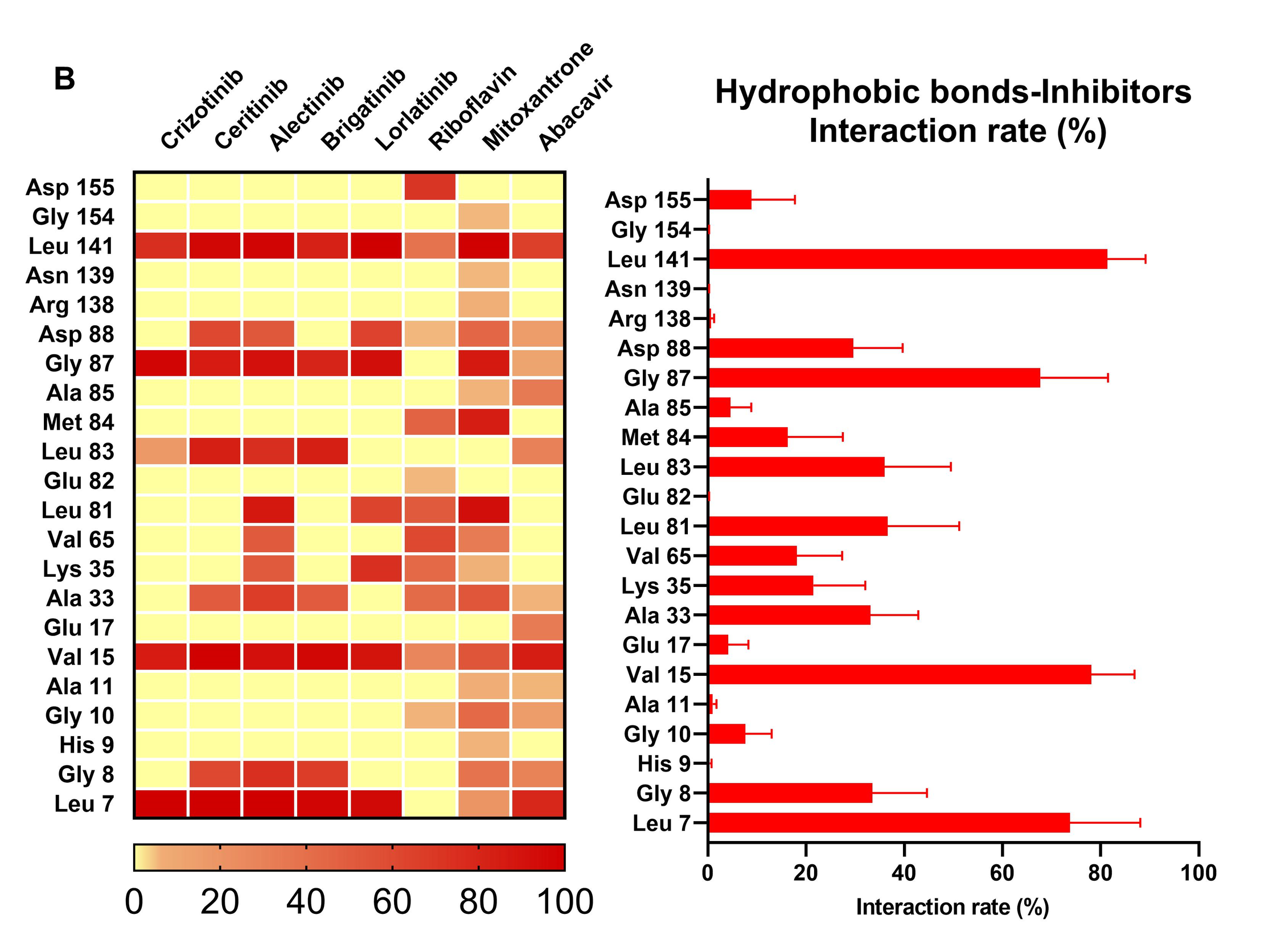

Supplement: S4 Fig — A) shows the aminoacids that have hydrogen bond interaction with the drugs under study, in addition to the rate throughout all the dynamics. B) shows the amino acids that have hydrophobic bond interactions with the drugs under study, in addition to the rate throughout all the dynamics. The yellow squares are amino acid that are not present in the interactions with iALK or FDA-approved drugs. (ZIP) [file pone.0295966.s006.zip › S4B_Fig.tif]

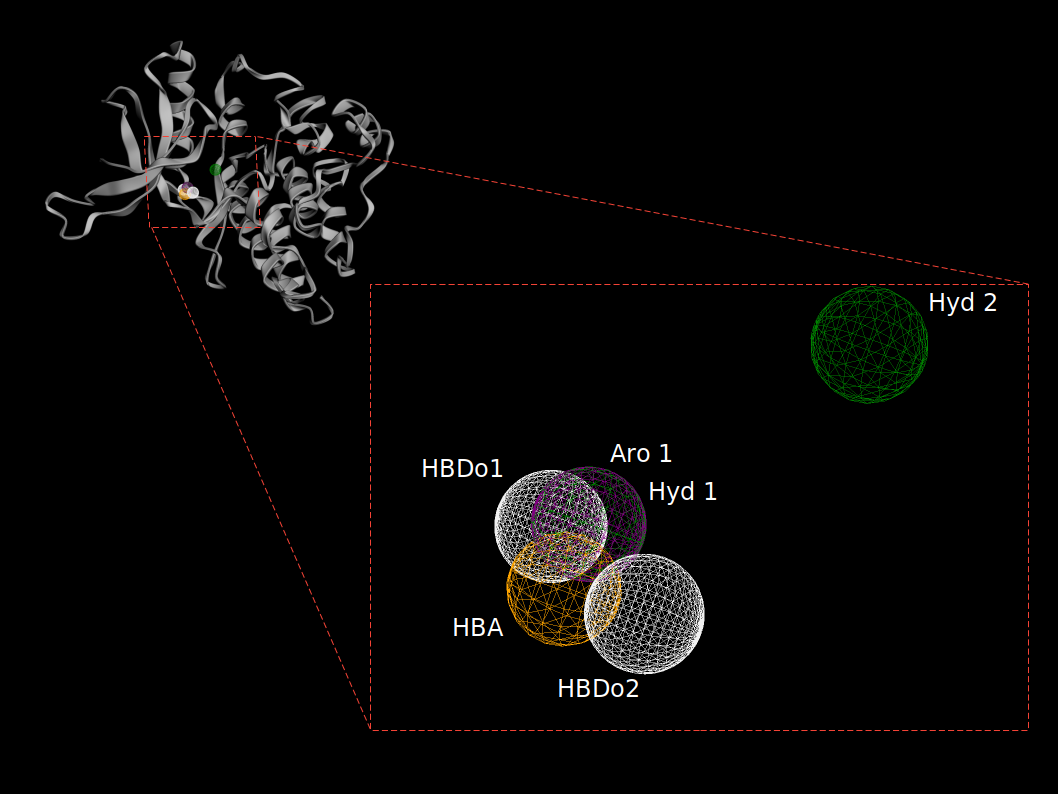

Supplement: S5 Fig — HBDo: hydrogen bond donor, HBA: hydrogen bond aceptor, Hyd: hydrophobic, Aro: aromatic. (TIF) [file pone.0295966.s007.tif]

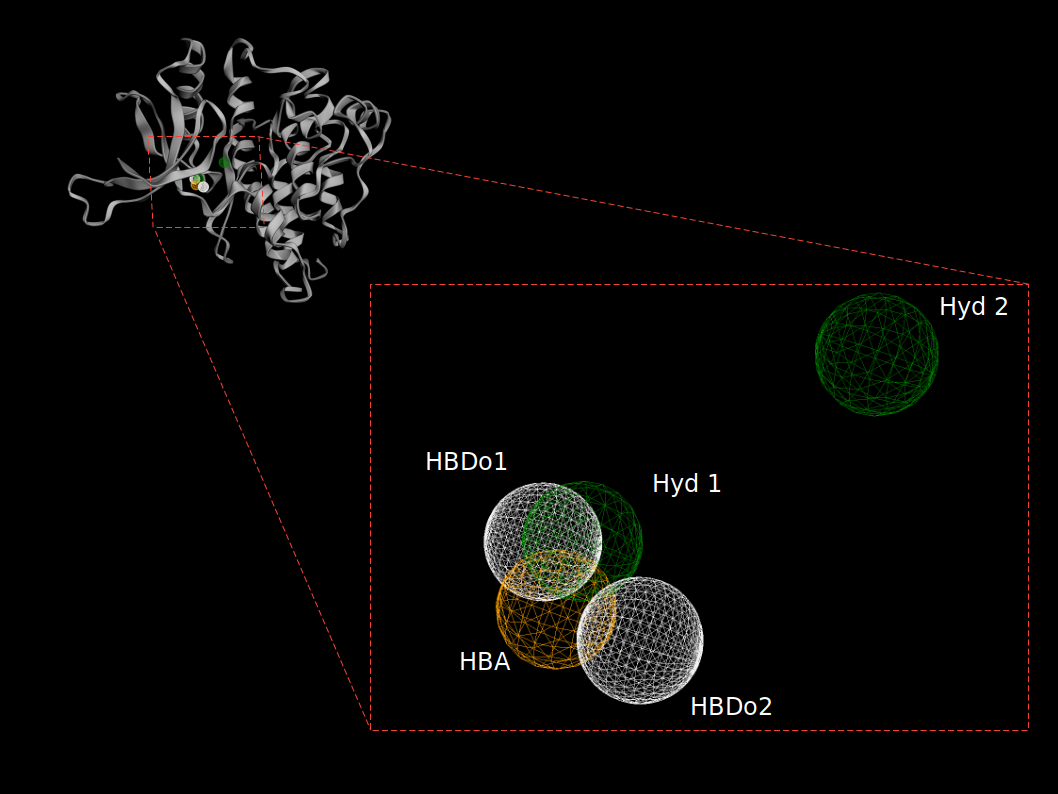

Supplement: S6 Fig — HBDo: hydrogen bond donor, HBA: hydrogen bond aceptor, Hyd: hydrophobic, Aro: aromatic. (TIF) [file pone.0295966.s008.tif]

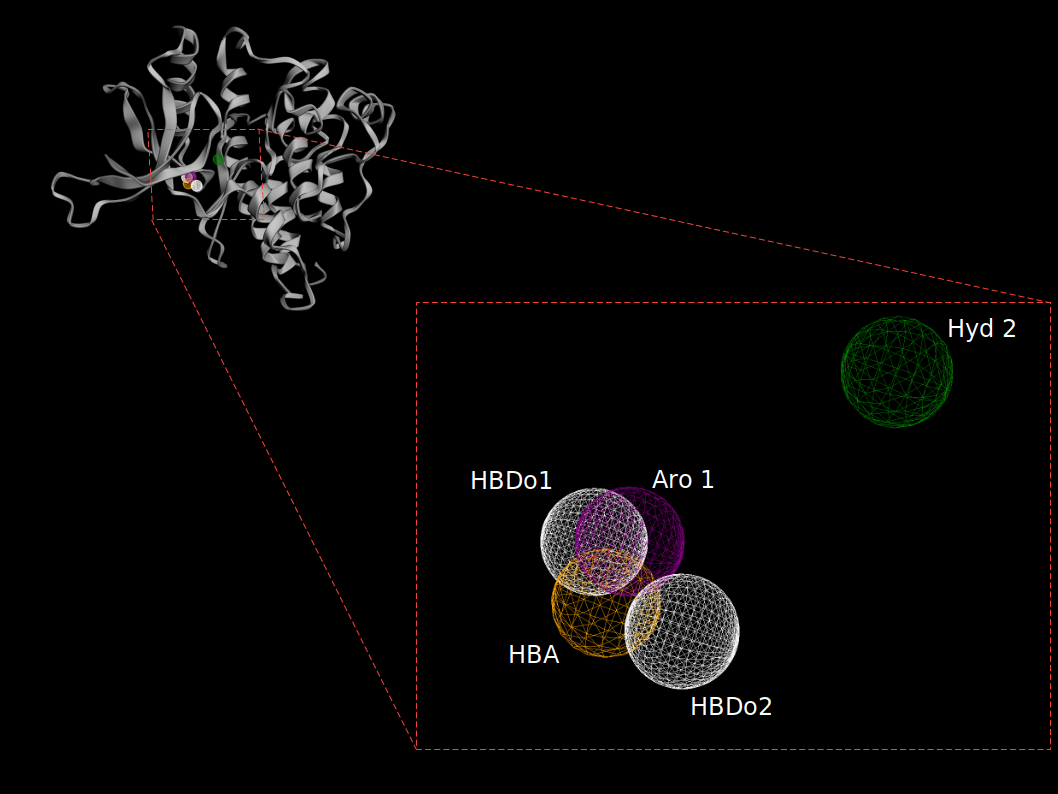

Supplement: S7 Fig — HBDo: hydrogen bond donor, HBA: hydrogen bond aceptor, Hyd: hydrophobic, Aro: aromatic. (TIF) [file pone.0295966.s009.tif]

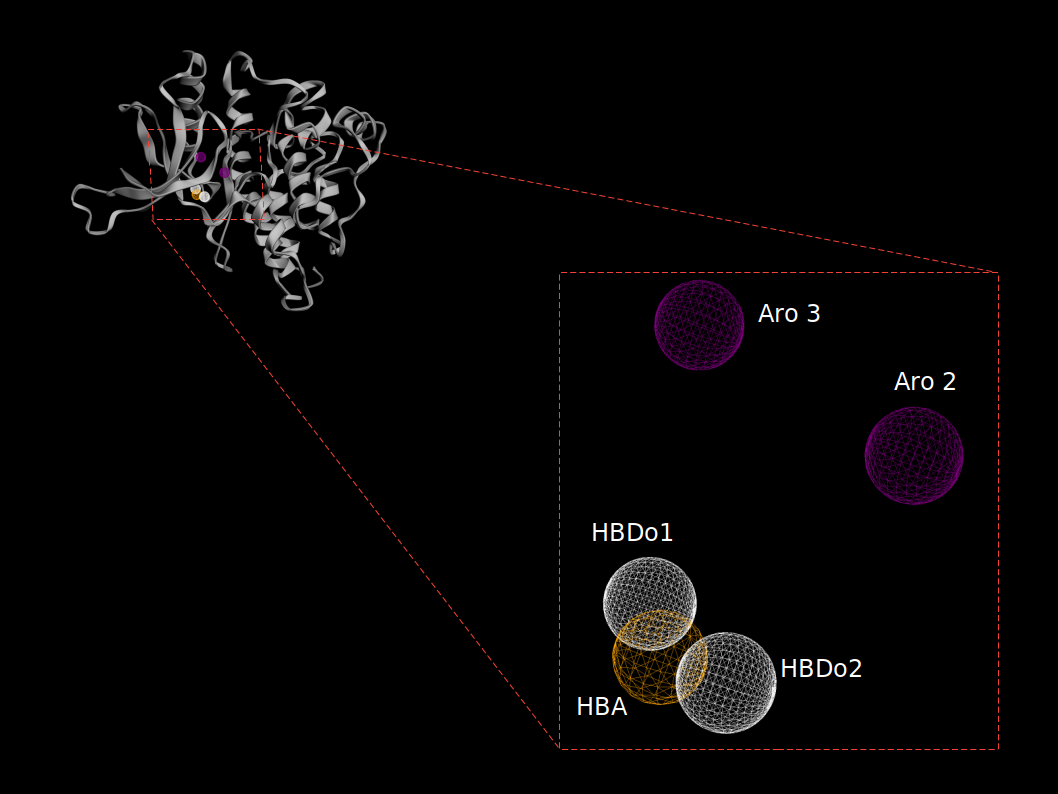

Supplement: S8 Fig — HBDo: hydrogen bond donor, HBA: hydrogen bond aceptor, Hyd: hydrophobic, Aro: aromatic. (TIF) [file pone.0295966.s010.tif]
