# Supplementary material for: Mitoxantrone and abacavir: An ALK protein-targeted in silico proposal for the treatment of non-small cell lung cancer
Source: PLoS One. 2024 Feb 6;19(2):e0295966. doi: 10.1371/journal.pone.0295966 (PMC10846704; doi:10.1371/journal.pone.0295966)
Supplement: Attachment — Submitted filename: comments.doc [file pone.0295966.s011.doc]

**Recommendation: Minor Revision**

**Comments:**

\# The abstract should be improved to clearly featured the aim and novelty of the work, the abstract may be on paragraph not sections

\# Why are these compounds chosen?

\# The reason for specific select of the PDB code: 5AAA, 4MKC, 3AOX, 6MX8 and 5AA9 should be given

\# For the Molecular Docking methodology, results, and discussions sections should be supported by more recently related Molecular Docking published works ; https://doi.org/10.3390/ijms232415614 ; https://doi.org/10.1016/j.molstruc.2022.134887 ; https://doi.org/10.1134/S1070363222120222 ; https://doi.org/10.3390/ma16030897 ; https://doi.org/10.3390/life13040912 ; https://doi.org/10.1134/S1070363223020214 ; https://doi.org/10.1002/aoc.6868 ; https://doi.org/10.1016/j.molliq.2022.120936

\# For Molecular dynamic ; Methodology should be clearly descried and used software’s and 4 force field, time, and ………….. should be clearly descried

#The structures of used FDA-approved drugs for Pharmacphore search may be added as supplementary material

\# A list of abbreviation used in the manuscript should be added

\# More values and comparisons may be added to conclusion part for improvement

\# A comparison between the current results and previous ones will improve the quality of the work
