# Supplementary material for: Mitoxantrone and abacavir: An ALK protein-targeted in silico proposal for the treatment of non-small cell lung cancer
Source: PLoS One. 2024 Feb 6;19(2):e0295966. doi: 10.1371/journal.pone.0295966 (PMC10846704; doi:10.1371/journal.pone.0295966)
Supplement: Attachment — Submitted filename: Response to Reviewers.docx [file pone.0295966.s012.docx]

**Rebuttal letter**

September 17, 2023

Dear Editor of PlosOne,

I hope this letter finds you well. I wish to express my gratitude for the opportunity to submit our article titled "Mitoxantrone and Abacavir: an ALK protein-targeted *in silico* proposal for the treatment of non-small cell lung cancer" by Faya Castillo *et al.* for consideration in PLOS ONE journal. I sincerely appreciate the time and resources that your editorial team has dedicated in evaluating our work.

Upon careful consideration of the comments and feedback provided by the expert reviewers and your editorial team regarding our article, I would like to address the queries and recommendations made during the review of the original manuscript. I hope that this response will aid in the reconsideration of the current decision regarding the acceptance of our article.

1. With regard to the formatting requirements specified by PLOS ONE, we have made revisions to the presentation of images, tables, and author affiliations in the revised version. The changes implemented in the revised manuscript are visually represented in the document titled "Revised Manuscript with Track Changes." Deletions have been clearly marked with yellow underlining, while additions are indicated with green underlining.
2. We have taken into consideration the option of providing the molecular dynamics results in ".dat" format for both cdALK^+^ and its associated complexes, including cdALK^+^-ATP, cdALK^+^-Crizotinib, cdALK^+^-Ceritinib, cdALK^+^-Brigatinib, cdALK^+^-Alectinib, cdALK^+^-Lorlatinib, cdALK^+^-Riboflavin, cdALK^+^-Abacavir, and cdALK^+^-Mitoxantrone. These data files are accessible on Dryad and can be downloaded via the provided link: <https://datadryad.org/stash/share/l2PCT3kGXyN6v_RlyY2BYlhCU4SGKN4CBWnpHS0yXfw>

**Regarding our responses to the reviewers' comments:**

**Reviewer #1**

The reviewer emphasized the significance of our research project but noted grammatical errors and the presence of lengthy, complex sentences that hindered comprehension. As a result, we have diligently revised the grammar, corrected errors, and restructured sentences to enhance readability for our readers.

In response to the reviewer's initial inquiry regarding the potential development of resistance with the proposed drugs and the applicability of molecular dynamics to understand this phenomenon:

In our article, we introduce riboflavin, abacavir, and mitoxantrone as potential inhibitors for non-small cell lung cancer, specifically when the catalytic domain of ALK is not mutated at the amino acid level. Molecular Dynamics enables us to approximate the emergence of resistance, primarily through the introduction of point mutations within the protein structure. However, due to our limited computational resources, conducting extensive dynamics simulations for all the mutations mentioned in the article by R. Bayliss et al. (2015), titled "Molecular mechanisms that underlie EML4-ALK-driven cancers and their response to targeted drugs," is not feasible.

To comprehensively address this concern, our laboratory plans to experimentally investigate the development of resistance to the proposed drugs. We intend to use the Ba/F3 EML4-ALKG1202R and Ba/F3 EML4-ALKL1196M cell lines as part of an ongoing project funded by CONCYTEC-PROCIENCIA under the Basic Research Projects call for 2019-01. Nonetheless, we firmly believe that the drugs proposed in our research possess the characteristics necessary for consideration as pharmacological repositioning agents, thereby contributing significantly to the understanding of NSCLC.

Furthermore, with regard to the reviewer's comments outlined below, we have addressed them in the revised manuscript as follows:

- We have prominently included the names of the drugs proposed for pharmacological repositioning at the beginning of the conclusion (Lines 396-397).
- We have provided a comprehensive explanation as to why riboflavin would not be considered a suitable candidate for pharmacological repositioning (Lines 403-409).
- We have rectified and standardized abbreviations throughout the entire manuscript.
- We have rephrased suggested sentences to enhance clarity and comprehension.

**Reviewer #2:**

We have duly considered the feedback from the second reviewer, resulting in the following actions taken in the revised manuscript:

- The abstract has been enhanced and condensed into a single paragraph, aligning with the reviewer's recommendation.
- An explanation has been included in the article, elucidating the rationale behind the selection of the compounds and the reasons for choosing specific PDB codes (Lines 151-153).
- Supplementary material (S2 Fig) has been updated to incorporate the structures of the drugs approved to generate the pharmacophore.
- Although there is no specific section for a list of abbreviations within the PLOS ONE manuscript format, we have included a separate document in ".doc" format with the requested abbreviations, which has been uploaded alongside other required files.
- Additional information has been incorporated into the conclusions section, offering further support for the utilization of the proposed drugs in the treatment of NSCLC.

I trust that this letter will serve as a useful resource in gaining a better understanding of my perspective and in addressing any concerns that may have arisen during the review process.

We firmly contend that this manuscript is well-suited for publication in the esteemed PLOS ONE journal. It not only introduces two novel drugs for pharmacological repositioning but also pushes the boundaries of scientific knowledge in the field of cancer. By doing so, it aligns seamlessly with PLOS ONE's mission to advance scientific understanding for the betterment of society.

I sincerely appreciate your time and attention to this matter and look forward to the possibility of having my article reconsidered for publication in PLOS ONE journal.

Sincerely,

Stefany Fiorella Infante Varillas

Medicine Faculty, University of Piura
Calle Mártir José Olaya 162, Miraflores 15074

[stefany.infante@udep.edu.pe](mailto:stefany.infante@udep.edu.pe)
