# Supplementary material for: Mitoxantrone and abacavir: An ALK protein-targeted in silico proposal for the treatment of non-small cell lung cancer
Source: PLoS One. 2024 Feb 6;19(2):e0295966. doi: 10.1371/journal.pone.0295966 (PMC10846704; doi:10.1371/journal.pone.0295966)
Supplement: Attachment — Submitted filename: Response to Reviewers.docx [file pone.0295966.s013.docx]

**Rebuttal letter**

November 30, 2023

Dear Editor of PlosOne,

I hope this letter finds you well. I wish to express my gratitude for the opportunity to submit our article titled "Mitoxantrone and Abacavir: an ALK protein-targeted *in silico* proposal for the treatment of non-small cell lung cancer" by Faya Castillo *et al.* for consideration in PLOS ONE journal. I sincerely appreciate the time and resources that your editorial team has dedicated in evaluating our work.

After careful consideration of the comments and opinions provided by the expert reviewers and your editorial team regarding our article, I would like to address the questions and recommendations raised during the review of the original manuscript. I trust that this response will contribute to a reconsideration of the current decision regarding the acceptance of our article.

With respect to the format requirements specified by PLOS ONE, everything is in order. The changes implemented in the revised manuscript are visually represented in red in the document titled 'Revised Manuscript with Track Changes.' Upon reviewing our script for the new experiments, we observed that the values obtained for binding energy in the molecular dockings should be represented in kJoul/mol; in Fig. 1 we change the unit to KJoul/mol. Therefore, we have made this change in the article without altering our results or discussions. Additionally, we have considered sharing the results from our initial molecular dynamics alongside the new molecular dynamics with mutated.

The list of molecular dynamics: cdALK^+^, cdALK^+^-ATP, cdALK^+^-Crizotinib, cdALK^+^-Ceritinib, cdALK^+^-Brigatinib, cdALK^+^-Alectinib, cdALK^+^-Lorlatinib, cdALK^+^-Riboflavin, cdALK^+^-Abacavir, cdALK^+^-Mitoxantrone, cdALK-ABA^L1196M^, cdALK-ABA^C1156Y^, cdALK-ABA^G1202R^, cdALK-MTX^L1196M^, cdALK-MTX^C1156Y^, and cdALK-MTX^G1202R^. These data files are accessible on Dryad and can be downloaded via the provided link: <https://datadryad.org/stash/share/l2PCT3kGXyN6v_RlyY2BYlhCU4SGKN4CBWnpHS0yXfw>

**Regarding our responses to the reviewers' comments:**

**Reviewer #1**

*In response to the reviewer's initial inquiry regarding the potential development of resistance with the proposed drugs and the applicability of molecular dynamics to understand this phenomenon:*

1. Can resistance also develop for the abacavir and mitoxantrone like other iALKs?

Following the first reviewer's recommendation, we conducted six 100 ns molecular dynamics simulations of mutated positions in cdALK: L1196M (resistant to crizotinib), C1156Y (resistant to crizotinib and alectinib), and G1202R (resistant to crizotinib, ceritinib, and alectinib). Subsequently, we generated minimized molecular docking complexes: cdALK^L1196M^-ABA, cdALK ^C1156Y^-ABA, cdALK^G1202R^-ABA, cdALK^L1196M^-MTX, cdALK^C1156Y^-MTX, and cdALK^G1202R^-MTX. These dynamics allowed us to computationally approximate the potential emergence of resistance against abacavir and mitoxantrone. The obtained results are more than interesting and are attached in the file named ***“Response to reviewer 1 - Resistance Approach”***. In summary, we believe that, despite the present mutations, the catalytic domain of ALK remains constant throughout the molecular dynamic time, similar to the cdALK^+^-ATP and cdALK^+^-ABA complexes, as well as the cdALK-ATP and cdALK^+^-MTX complexes. This suggests that abacavir and mitoxantrone might have an effect on cdALK despite the mutations. We have observed only a slight difference in the ALK-ABA^C1156Y^ complex, which is explained in the attached document.

For this reason, more than enthusiastic about the results, we will initiate, as part of the project funded by CONCYTEC-PROCIENCIA under the Basic Research Projects call for 2019-01, an *in silico* and *in vitro* study related to the possibility of resistance that ALK may present to the drugs abacavir (ABA) and mitoxantrone (MTX). This will involve the use of the cell lines Ba/F3 EML4-ALK^L1196M^, Ba/F3 EML4-ALK^C1156Y^, and Ba/F3 EML4-ALK^G1202R^. These cell models of non-small cell lung cancer (Ba/F3 EML4-ALK^WT^, Ba/F3 EML4-ALK^L1196M^, Ba/F3 EML4-ALK^C1156Y^ and Ba/F3 EML4-ALK^G1202R^) were generated by Site-directed mutagenesis by Fontana D. et al, and kindly provided for this future research.

2. Can a MD simulation study prove that resistance generation for these molecules will be minimized?

The answer to this can be addressed by referencing Priya Doss *et al*.^1^, who employs bioinformatics methods to predict the impact of mutations on ALK. The initial approach of that project involves point mutations using the Swiss-PdbViewer program on the ALK crystal stored in the PDB (ID: 3L9P), followed by energy minimization, generating the F1174L and R1275Q mutations. Then, the structure was docked with native ALK, ALK-F1174L, and ALK-R1275Q, followed by molecular dynamics simulations of the complexes. The conclusion was drawn that F1174L could have a significant impact on protein function, aligning with the results obtained by Mossé *et al*.^2^.

On the other hand, molecular dynamics studies with a focus on determining whether point mutations lead to resistance to a respective drug have been frequently employed. Jian Li *et al*.^3^, for instance, addressed the possibility that the L1198F mutation resensitizes ALK to interact again with crizotinib, concluding that this acquired resistance by L1198F causes conformational changes in the ATP-binding sites of the ALK protein, which could enable resensitization to crizotinib.

Finally, works like that of Wang *et al*.^4^ highlight the effectiveness of molecular dynamics and related techniques in understanding mutation-induced diseases, thereby allowing for the more efficient development of innovative drugs.

1. Priya G, Chakraborty C, Chen l, Zhu H. Integrating *In Silico* Prediction Methods, Molecular Docking, and Molecular Dynamics Simulation to Predict the Impact of ALK Missense Mutations in Structural Perspective. BioMed Research International. 2014; 2014, 1-14. doi: [10.1155/2014/895831](https://doi.org/10.1155/2014/895831)

2. Mossé Y, Laudenslager M, Longo L. et al. Identification of ALK as a major familial neuroblastoma predisposition gene. 2008. Nature. 455; 930–935. Doi: 10.1038/nature07261

3. Li J, Sun R, Wu Y, Song M, Li J, Yang Q, Chen X, Bao J, Zhao Q. L1198F Mutation Resensitizes Crizotinib to ALK by Altering the Conformation of Inhibitor and ATP Binding Sites. 2017. Int J Mol Sci. 18 482-494. doi: 10.3390/ijms18030482.

4. Wang D, Ou-Yang L, Xie H, Zhu M, Yan H. Predicting the impacts of mutations on protein-ligand binding affinity based on molecular dynamics simulations and machine learning methods. 2020. Computational and Structural Biotechnology Journal. 2020; 18:439–454. doi: [10.1016/j.csbj.2020.02.007](https://doi.org/10.1016/j.csbj.2020.02.007)

We firmly contend that this manuscript is well-suited for publication in the esteemed PLOS ONE journal. It not only introduces two novel drugs for pharmacological repositioning but also pushes the boundaries of scientific knowledge in the field of cancer. By doing so, it aligns seamlessly with PLOS ONE's mission to advance scientific understanding for the betterment of society.

I sincerely appreciate your time and attention, and I look forward to the possibility of our article being considered for publication in PLOS ONE. This would mark our research group's first publication.

Sincerely,

Stefany Fiorella Infante Varillas

Medicine Faculty, University of Piura
Calle Mártir José Olaya 162, Miraflores 15074

[stefany.infante@udep.edu.pe](mailto:stefany.infante@udep.edu.pe)
