# Supplementary material for: Mitoxantrone and abacavir: An ALK protein-targeted in silico proposal for the treatment of non-small cell lung cancer
Source: PLoS One. 2024 Feb 6;19(2):e0295966. doi: 10.1371/journal.pone.0295966 (PMC10846704; doi:10.1371/journal.pone.0295966)
Supplement: Peer review history

# Peer review: Decision Letter 0

**DOI:** 10.1371/journal.pone.0295966.r001

**Authors:** Chandrabose Selvaraj

3 Aug 2023

PONE-D-23-20740Mitoxantrone and Abacavir: an ALK protein-targeted in silico proposal for the treatment of non-small cell lung cancer.PLOS ONE

Dear Dr. Faya,

Thank you for submitting your manuscript to PLOS ONE. After careful consideration, we feel that it has merit but does not fully meet PLOS ONE’s publication criteria as it currently stands. Therefore, we invite you to submit a revised version of the manuscript that addresses the points raised during the review process.

Please submit your revised manuscript by Sep 17 2023 11:59PM. If you will need more time than this to complete your revisions, please reply to this message or contact the journal office at plosone@plos.org. When you're ready to submit your revision, log on to https://www.editorialmanager.com/pone/ and select the 'Submissions Needing Revision' folder to locate your manuscript file.

Please include the following items when submitting your revised manuscript:

- A rebuttal letter that responds to each point raised by the academic editor and reviewer(s). You should upload this letter as a separate file labeled 'Response to Reviewers'.
- A marked-up copy of your manuscript that highlights changes made to the original version. You should upload this as a separate file labeled 'Revised Manuscript with Track Changes'.
- An unmarked version of your revised paper without tracked changes. You should upload this as a separate file labeled 'Manuscript'.

If you would like to make changes to your financial disclosure, please include your updated statement in your cover letter. Guidelines for resubmitting your figure files are available below the reviewer comments at the end of this letter.

If applicable, we recommend that you deposit your laboratory protocols in protocols.io to enhance the reproducibility of your results. Protocols.io assigns your protocol its own identifier (DOI) so that it can be cited independently in the future. For instructions see: https://journals.plos.org/plosone/s/submission-guidelines#loc-laboratory-protocols. Additionally, PLOS ONE offers an option for publishing peer-reviewed Lab Protocol articles, which describe protocols hosted on protocols.io. Read more information on sharing protocols at https://plos.org/protocols?utm_medium=editorial-email&utm_source=authorletters&utm_campaign=protocols.

We look forward to receiving your revised manuscript.

Kind regards,

Chandrabose Selvaraj, Ph.D.

Academic Editor

PLOS ONE

Journal Requirements:

When submitting your revision, we need you to address these additional requirements.

1. Please ensure that your manuscript meets PLOS ONE's style requirements, including those for file naming. The PLOS ONE style templates can be found at 

https://journals.plos.org/plosone/s/file?id=wjVg/PLOSOne_formatting_sample_main_body.pdf and 

https://journals.plos.org/plosone/s/file?id=ba62/PLOSOne_formatting_sample_title_authors_affiliations.pdf

2. Please note that PLOS ONE has specific guidelines on code sharing for submissions in which author-generated code underpins the findings in the manuscript. In these cases, all author-generated code must be made available without restrictions upon publication of the work. Please review our guidelines at https://journals.plos.org/plosone/s/materials-and-software-sharing#loc-sharing-code and ensure that your code is shared in a way that follows best practice and facilitates reproducibility and reuse.

3. We note that you have indicated that data from this study are available upon request. PLOS only allows data to be available upon request if there are legal or ethical restrictions on sharing data publicly. For more information on unacceptable data access restrictions, please see http://journals.plos.org/plosone/s/data-availability#loc-unacceptable-data-access-restrictions. 

In your revised cover letter, please address the following prompts:

a) If there are ethical or legal restrictions on sharing a de-identified data set, please explain them in detail (e.g., data contain potentially sensitive information, data are owned by a third-party organization, etc.) and who has imposed them (e.g., an ethics committee). Please also provide contact information for a data access committee, ethics committee, or other institutional body to which data requests may be sent.

b) If there are no restrictions, please upload the minimal anonymized data set necessary to replicate your study findings as either Supporting Information files or to a stable, public repository and provide us with the relevant URLs, DOIs, or accession numbers. For a list of acceptable repositories, please see http://journals.plos.org/plosone/s/data-availability#loc-recommended-repositories.

We will update your Data Availability statement on your behalf to reflect the information you provide.

Additional Editor Comments:

One or more of the reviewers has recommended that you cite specific previously published works. The works referenced are not directly related to the submitted manuscript. As such, please note that it is not necessary or expected to cite the works requested by the reviewer.

[Note: HTML markup is below. Please do not edit.]

Reviewers' comments:

Reviewer's Responses to Questions

**Comments to the Author**

1. Is the manuscript technically sound, and do the data support the conclusions?

The manuscript must describe a technically sound piece of scientific research with data that supports the conclusions. Experiments must have been conducted rigorously, with appropriate controls, replication, and sample sizes. The conclusions must be drawn appropriately based on the data presented. 

Reviewer #1: Yes

Reviewer #2: Yes

**********

2. Has the statistical analysis been performed appropriately and rigorously? 

Reviewer #1: Yes

Reviewer #2: Yes

**********

3. Have the authors made all data underlying the findings in their manuscript fully available?

The PLOS Data policy requires authors to make all data underlying the findings described in their manuscript fully available without restriction, with rare exception (please refer to the Data Availability Statement in the manuscript PDF file). The data should be provided as part of the manuscript or its supporting information, or deposited to a public repository. For example, in addition to summary statistics, the data points behind means, medians and variance measures should be available. If there are restrictions on publicly sharing data—e.g. participant privacy or use of data from a third party—those must be specified.

Reviewer #1: Yes

Reviewer #2: Yes

**********

4. Is the manuscript presented in an intelligible fashion and written in standard English?

PLOS ONE does not copyedit accepted manuscripts, so the language in submitted articles must be clear, correct, and unambiguous. Any typographical or grammatical errors should be corrected at revision, so please note any specific errors here.

Reviewer #1: No

Reviewer #2: Yes

**********

5. Review Comments to the Author

Please use the space provided to explain your answers to the questions above. You may also include additional comments for the author, including concerns about dual publication, research ethics, or publication ethics. (Please upload your review as an attachment if it exceeds 20,000 characters)

Reviewer #1: The research aimed at repurposing drugs for ALK inhibition in lung cancer. The current findings of the work are good and useful.

The entire text has grammar and language related issues that need to be corrected for acceptance. Some sentences are too long and are difficult to understand. Also, there are several spelling mistakes.

1. Can resistance also develop for the ribloflavin, abacavir and mitoxantrone like other iALKs? Can a MD simulation study prove that resistance generation for these molecules will be minimized?

2. In conclusion, which three drugs showed good activity should be mentioned clearly in the beginning of the paragraph (Line 390).

3. The binding energy of abacavir is less than riboflavin, still the author have concluded in the conclusion section that they propose metoxantrone and abacavir as more suitable candidate for inhibition of ALK and not riboflavin. This shall be explained.

4. Abbreviations need to be corrected in the entire manuscript. Words in their full form are repeated instead of abbreviations or otherwise abbreviated at their latter usage and not first usage.

5. Line 40-41: Kindly reframe the sentence for clear understanding.

6. Line 43-47: Reframe the sentence and make it short.

7. Line 47-49: Reframe the sentence.

8. Figure S1 and S2 must be labelled. Glu82 and Met84, part of the ALK hinge

318 region together with Leu-83, Ala85 and Gly86 must be highlighted or labelled. Ligand interaction diagram showing a closer look at how these residues interact with the ligand must be added.

9. Table 2, S1: numbers are separated by commas. Correct them.

10. Line 148-149: The binding energy value is expressed numbers separated by commas. Correct them.

11. Reframe Line 299-304 for more clarity.

12. Headings and subheadings should be numbered in the entire manuscript.

Reviewer #2: Recommendation: Minor Revision

Comments:

# The abstract should be improved to clearly featured the aim and novelty of the work, the abstract may be on paragraph not sections

# Why are these compounds chosen?

# The reason for specific select of the PDB code: 5AAA, 4MKC, 3AOX, 6MX8 and 5AA9 should be given

# For the Molecular Docking methodology, results, and discussions sections should be supported by more recently related Molecular Docking published works ; https://doi.org/10.3390/ijms232415614 ; https://doi.org/10.1016/j.molstruc.2022.134887 ; https://doi.org/10.1134/S1070363222120222 ; https://doi.org/10.3390/ma16030897 ; https://doi.org/10.3390/life13040912 ; https://doi.org/10.1134/S1070363223020214 ; https://doi.org/10.1002/aoc.6868 ; https://doi.org/10.1016/j.molliq.2022.120936

# For Molecular dynamic ; Methodology should be clearly descried and used software’s and 4 force field, time, and ………….. should be clearly descried

#The structures of used FDA-approved drugs for Pharmacophore search may be added as supplementary material

# A list of abbreviation used in the manuscript should be added

# More values and comparisons may be added to conclusion part for improvement

# A comparison between the current results and previous ones will improve the quality of the work

**********

6. PLOS authors have the option to publish the peer review history of their article (what does this mean?). If published, this will include your full peer review and any attached files.

If you choose “no”, your identity will remain anonymous but your review may still be made public.

**Do you want your identity to be public for this peer review?** For information about this choice, including consent withdrawal, please see our Privacy Policy.

Reviewer #1: No

Reviewer #2: No

**********

[NOTE: If reviewer comments were submitted as an attachment file, they will be attached to this email and accessible via the submission site. Please log into your account, locate the manuscript record, and check for the action link "View Attachments". If this link does not appear, there are no attachment files.]

While revising your submission, please upload your figure files to the Preflight Analysis and Conversion Engine (PACE) digital diagnostic tool, https://pacev2.apexcovantage.com/. PACE helps ensure that figures meet PLOS requirements. To use PACE, you must first register as a user. Registration is free. Then, login and navigate to the UPLOAD tab, where you will find detailed instructions on how to use the tool. If you encounter any issues or have any questions when using PACE, please email PLOS at figures@plos.org. Please note that Supporting Information files do not need this step.

Attachment Submitted filename: comments.doc Click here for additional data file.

# Author comment: Author response to Decision Letter 0

**DOI:** 10.1371/journal.pone.0295966.r002

17 Sep 2023

Editor: 

1. With regard to the formatting requirements specified by PLOS ONE, we have made revisions to the presentation of images, tables, and author affiliations in the revised version.

2. The codes utilized in this article are adaptations of the scripts initially provided by the YASARA program under its licensing agreement. However, if required, these scripts can be located and customized at the following web address: http://www.yasara.org/macros.htm. For our project, we have specifically employed the md_run.mcr script.

3. 2. We have taken into consideration the option of providing the molecular dynamics results in ".dat" format for both cdALK+ and its associated complexes, including cdALK+-ATP, cdALK+-Crizotinib, cdALK+-Ceritinib, cdALK+-Brigatinib, cdALK+-Alectinib, cdALK+-Lorlatinib, cdALK+-Riboflavin, cdALK+-Abacavir, and cdALK+-Mitoxantrone. These data files are accessible on Dryad and can be downloaded via the provided link: https://datadryad.org/stash/share/l2PCT3kGXyN6v_RlyY2BYlhCU4SGKN4CBWnpHS0yXfw

Regarding our responses to the reviewers' comments:

Reviewer #1

1. In our article, we introduce riboflavin, abacavir, and mitoxantrone as potential inhibitors for non-small cell lung cancer, specifically when the catalytic domain of ALK is not mutated at the amino acid level. Molecular Dynamics enables us to approximate the emergence of resistance, primarily through the introduction of point mutations within the protein structure. However, due to our limited computational resources, conducting extensive dynamics simulations for all the mutations mentioned in the article by R. Bayliss et al. (2015), titled "Molecular mechanisms that underlie EML4-ALK-driven cancers and their response to targeted drugs," is not feasible.

To comprehensively address this concern, our laboratory plans to experimentally investigate the development of resistance to the proposed drugs. We intend to use the Ba/F3 EML4-ALKG1202R and Ba/F3 EML4-ALKL1196M cell lines as part of an ongoing project funded by CONCYTEC-PROCIENCIA under the Basic Research Projects call for 2019-01. Nonetheless, we firmly believe that the drugs proposed in our research possess the characteristics necessary for consideration as pharmacological repositioning agents, thereby contributing significantly to the understanding of NSCLC.

2. We have prominently included the names of the drugs proposed for pharmacological repositioning at the beginning of the conclusion (Lines 396-397).

3. Although riboflavin exhibits superior binding energy to ATP, previous studies demonstrated its adverse activity by increasing cell proliferation and migration in A549 and H3255 non-small cell lung cancer cell models. Hence, riboflavin does not appear to be a promising candidate for the suggested pharmacological repositioning. However, the structural attributes shared between Riboflavin and the other two drugs offer a valuable foundation for the development of novel ATP-competitive anti-tumoral for ALK+NSCLC. Yan et al. (2013).

4. We have rectified and standardized abbreviations throughout the entire manuscript.

5 - 7, 11. We have rephrased suggested sentences to enhance clarity and comprehension.

8. Figures have been labeled, and critical amino acids have been distinctly highlighted.

9 - 10. Commas have been replaced by points.

12. Titles and subtitles were numbered throughout the manuscript.

Reviewer #2

1. The abstract has been enhanced and condensed into a single paragraph, aligning with the reviewer's recommendation.

2. The PDB templates were selected because they feature the structure of cdALK+ in association with specific inhibitors of CPCNP. However, these structures are incomplete, necessitating the use of molecular modeling to complete the structure.

3. For the Molecular Docking methodology, the proposed articles were taken into account.

4. The structures of FDA-approved drugs used for pharmacophore searching have been added as supplementary material (S2 Fig).

5. Although there is no specific section for a list of abbreviations within the PLOS ONE manuscript format, we have included a separate document in ".doc" format with the requested abbreviations, which has been uploaded alongside other required files.

6. Additional information has been incorporated into the conclusions section, offering further support for the utilization of the proposed drugs in the treatment of NSCLC.

Attachment Submitted filename: Response to Reviewers.docx Click here for additional data file.

# Peer review: Decision Letter 1

**DOI:** 10.1371/journal.pone.0295966.r003

**Authors:** Chandrabose Selvaraj

16 Oct 2023

PONE-D-23-20740R1Mitoxantrone and Abacavir: an ALK protein-targeted in silico proposal for the treatment of non-small cell lung cancer.PLOS ONE

Dear Dr. Faya,

Thank you for submitting your manuscript to PLOS ONE. After careful consideration, we feel that it has merit but does not fully meet PLOS ONE’s publication criteria as it currently stands. Therefore, we invite you to submit a revised version of the manuscript that addresses the points raised during the review process.

Please submit your revised manuscript by Nov 30 2023 11:59PM. If you will need more time than this to complete your revisions, please reply to this message or contact the journal office at plosone@plos.org. When you're ready to submit your revision, log on to https://www.editorialmanager.com/pone/ and select the 'Submissions Needing Revision' folder to locate your manuscript file.

Please include the following items when submitting your revised manuscript:

- A rebuttal letter that responds to each point raised by the academic editor and reviewer(s). You should upload this letter as a separate file labeled 'Response to Reviewers'.
- A marked-up copy of your manuscript that highlights changes made to the original version. You should upload this as a separate file labeled 'Revised Manuscript with Track Changes'.
- An unmarked version of your revised paper without tracked changes. You should upload this as a separate file labeled 'Manuscript'.

If you would like to make changes to your financial disclosure, please include your updated statement in your cover letter. Guidelines for resubmitting your figure files are available below the reviewer comments at the end of this letter.

If applicable, we recommend that you deposit your laboratory protocols in protocols.io to enhance the reproducibility of your results. Protocols.io assigns your protocol its own identifier (DOI) so that it can be cited independently in the future. For instructions see: https://journals.plos.org/plosone/s/submission-guidelines#loc-laboratory-protocols. Additionally, PLOS ONE offers an option for publishing peer-reviewed Lab Protocol articles, which describe protocols hosted on protocols.io. Read more information on sharing protocols at https://plos.org/protocols?utm_medium=editorial-email&utm_source=authorletters&utm_campaign=protocols.

We look forward to receiving your revised manuscript.

Kind regards,

Chandrabose Selvaraj, Ph.D.

Academic Editor

PLOS ONE

Journal Requirements:

Please review your reference list to ensure that it is complete and correct. If you have cited papers that have been retracted, please include the rationale for doing so in the manuscript text, or remove these references and replace them with relevant current references. Any changes to the reference list should be mentioned in the rebuttal letter that accompanies your revised manuscript. If you need to cite a retracted article, indicate the article’s retracted status in the References list and also include a citation and full reference for the retraction notice.

[Note: HTML markup is below. Please do not edit.]

Reviewers' comments:

Reviewer's Responses to Questions

**Comments to the Author**

1. If the authors have adequately addressed your comments raised in a previous round of review and you feel that this manuscript is now acceptable for publication, you may indicate that here to bypass the “Comments to the Author” section, enter your conflict of interest statement in the “Confidential to Editor” section, and submit your "Accept" recommendation.

Reviewer #1: All comments have been addressed

Reviewer #2: All comments have been addressed

**********

2. Is the manuscript technically sound, and do the data support the conclusions?

The manuscript must describe a technically sound piece of scientific research with data that supports the conclusions. Experiments must have been conducted rigorously, with appropriate controls, replication, and sample sizes. The conclusions must be drawn appropriately based on the data presented. 

Reviewer #1: Yes

Reviewer #2: Yes

**********

3. Has the statistical analysis been performed appropriately and rigorously? 

Reviewer #1: Yes

Reviewer #2: N/A

**********

4. Have the authors made all data underlying the findings in their manuscript fully available?

The PLOS Data policy requires authors to make all data underlying the findings described in their manuscript fully available without restriction, with rare exception (please refer to the Data Availability Statement in the manuscript PDF file). The data should be provided as part of the manuscript or its supporting information, or deposited to a public repository. For example, in addition to summary statistics, the data points behind means, medians and variance measures should be available. If there are restrictions on publicly sharing data—e.g. participant privacy or use of data from a third party—those must be specified.

Reviewer #1: Yes

Reviewer #2: Yes

**********

5. Is the manuscript presented in an intelligible fashion and written in standard English?

PLOS ONE does not copyedit accepted manuscripts, so the language in submitted articles must be clear, correct, and unambiguous. Any typographical or grammatical errors should be corrected at revision, so please note any specific errors here.

Reviewer #1: Yes

Reviewer #2: Yes

**********

6. Review Comments to the Author

Please use the space provided to explain your answers to the questions above. You may also include additional comments for the author, including concerns about dual publication, research ethics, or publication ethics. (Please upload your review as an attachment if it exceeds 20,000 characters)

Reviewer #1: The authors have addressed all other comments satisfactorily except the below comment.

8. Can resistance also develop for the ribloflavin, abacavir and mitoxantrone like other iALKs? Can a MD simulation study prove that resistance generation for these molecules will be minimized?

Authors have started cell based assays or are planning to perform is unclear.

Further, literature references shall be provided for the same.

Thank you.

Reviewer #2: Recommendation: Acceptance

Comments:

The authors improved their manuscript and answered all the asked quires and the article are now suitable for acceptance for publication in PLOS ONE journal

**********

7. PLOS authors have the option to publish the peer review history of their article (what does this mean?). If published, this will include your full peer review and any attached files.

If you choose “no”, your identity will remain anonymous but your review may still be made public.

**Do you want your identity to be public for this peer review?** For information about this choice, including consent withdrawal, please see our Privacy Policy.

Reviewer #1: No

Reviewer #2: No

**********

[NOTE: If reviewer comments were submitted as an attachment file, they will be attached to this email and accessible via the submission site. Please log into your account, locate the manuscript record, and check for the action link "View Attachments". If this link does not appear, there are no attachment files.]

While revising your submission, please upload your figure files to the Preflight Analysis and Conversion Engine (PACE) digital diagnostic tool, https://pacev2.apexcovantage.com/. PACE helps ensure that figures meet PLOS requirements. To use PACE, you must first register as a user. Registration is free. Then, login and navigate to the UPLOAD tab, where you will find detailed instructions on how to use the tool. If you encounter any issues or have any questions when using PACE, please email PLOS at figures@plos.org. Please note that Supporting Information files do not need this step.

# Author comment: Author response to Decision Letter 1

**DOI:** 10.1371/journal.pone.0295966.r004

1 Dec 2023

Reviewer #1

1.Can resistance also develop for the abacavir and mitoxantrone like other iALKs?

Following the first reviewer's recommendation, we conducted six 100 ns molecular dynamics simulations of mutated positions in cdALK: L1196M (resistant to crizotinib), C1156Y (resistant to crizotinib and alectinib), and G1202R (resistant to crizotinib, ceritinib, and alectinib). Subsequently, we generated minimized molecular docking complexes: cdALKL1196M-ABA, cdALK C1156Y-ABA, cdALKG1202R-ABA, cdALKL1196M-MTX, cdALKC1156Y-MTX, and cdALKG1202R-MTX. These dynamics allowed us to computationally approximate the potential emergence of resistance against abacavir and mitoxantrone. The obtained results are more than interesting and are attached in the file named 'Response to reviewer 1 - Resistance Approach'. In summary, we believe that, despite the present mutations, the catalytic domain of ALK remains constant throughout the molecular dynamic time, similar to the cdALK+-ATP and cdALK+-ABA complexes, as well as the cdALK-ATP and cdALK+-MTX complexes. This suggests that abacavir and mitoxantrone might have an effect on cdALK despite the mutations. We have observed only a slight difference in the ALK-ABAC1156Y complex, which is explained in the attached document. 

For this reason, more than enthusiastic about the results, we will initiate, as part of the project funded by CONCYTEC-PROCIENCIA under the Basic Research Projects call for 2019-01, an in silico and in vitro study related to the possibility of resistance that ALK may present to the drugs abacavir (ABA) and mitoxantrone (MTX). This will involve the use of the cell lines Ba/F3 EML4-ALKL1196M, Ba/F3 EML4-ALKC1156Y, and Ba/F3 EML4-ALKG1202R. These cell models of non-small cell lung cancer (Ba/F3 EML4-ALKWT, Ba/F3 EML4-ALKL1196M, Ba/F3 EML4-ALKC1156Y and Ba/F3 EML4-ALKG1202R) were generated by Site-directed mutagenesis by Fontana D. et al, and kindly provided for this future research.

2. Can a MD simulation study prove that resistance generation for these molecules will be minimized?

The answer to this can be addressed by referencing Priya Doss et al.1, who employs bioinformatics methods to predict the impact of mutations on ALK. The initial approach of that project involves point mutations using the Swiss-PdbViewer program on the ALK crystal stored in the PDB (ID: 3L9P), followed by energy minimization, generating the F1174L and R1275Q mutations. Then, the structure was docked with native ALK, ALK-F1174L, and ALK-R1275Q, followed by molecular dynamics simulations of the complexes. The conclusion was drawn that F1174L could have a significant impact on protein function, aligning with the results obtained by Mossé et al.2.

On the other hand, molecular dynamics studies with a focus on determining whether point mutations lead to resistance to a respective drug have been frequently employed. Jian Li et al.3, for instance, addressed the possibility that the L1198F mutation resensitizes ALK to interact again with crizotinib, concluding that this acquired resistance by L1198F causes conformational changes in the ATP-binding sites of the ALK protein, which could enable resensitization to crizotinib.

Finally, works like that of Wang et al.4 highlight the effectiveness of molecular dynamics and related techniques in understanding mutation-induced diseases, thereby allowing for the more efficient development of innovative drugs.

1. Priya G, Chakraborty C, Chen l, Zhu H. Integrating In Silico Prediction Methods, Molecular Docking, and Molecular Dynamics Simulation to Predict the Impact of ALK Missense Mutations in Structural Perspective. BioMed Research International. 2014; 2014, 1-14. doi: 10.1155/2014/895831

2. Mossé Y, Laudenslager M, Longo L. et al. Identification of ALK as a major familial neuroblastoma predisposition gene. 2008. Nature. 455; 930–935. Doi: 10.1038/nature07261

3. Li J, Sun R, Wu Y, Song M, Li J, Yang Q, Chen X, Bao J, Zhao Q. L1198F Mutation Resensitizes Crizotinib to ALK by Altering the Conformation of Inhibitor and ATP Binding Sites. 2017. Int J Mol Sci. 18 482-494. doi: 10.3390/ijms18030482.

4. Wang D, Ou-Yang L, Xie H, Zhu M, Yan H. Predicting the impacts of mutations on protein-ligand binding affinity based on molecular dynamics simulations and machine learning methods. 2020. Computational and Structural Biotechnology Journal. 2020; 18:439–454. doi: 10.1016/j.csbj.2020.02.007

Attachment Submitted filename: Response to Reviewers.docx Click here for additional data file.

# Editor report: Decision Letter 2

**DOI:** 10.1371/journal.pone.0295966.r005

**Authors:** Chandrabose Selvaraj

4 Dec 2023

Mitoxantrone and Abacavir: an ALK protein-targeted in silico proposal for the treatment of non-small cell lung cancer.

PONE-D-23-20740R2

Dear Dr. Faya,

We’re pleased to inform you that your manuscript has been judged scientifically suitable for publication and will be formally accepted for publication once it meets all outstanding technical requirements.

Within one week, you’ll receive an e-mail detailing the required amendments. When these have been addressed, you’ll receive a formal acceptance letter and your manuscript will be scheduled for publication.

An invoice for payment will follow shortly after the formal acceptance. To ensure an efficient process, please log into Editorial Manager at http://www.editorialmanager.com/pone/, click the 'Update My Information' link at the top of the page, and double check that your user information is up-to-date. If you have any billing related questions, please contact our Author Billing department directly at authorbilling@plos.org.

If your institution or institutions have a press office, please notify them about your upcoming paper to help maximize its impact. If they’ll be preparing press materials, please inform our press team as soon as possible -- no later than 48 hours after receiving the formal acceptance. Your manuscript will remain under strict press embargo until 2 pm Eastern Time on the date of publication. For more information, please contact onepress@plos.org.

Kind regards,

Chandrabose Selvaraj, Ph.D.

Academic Editor

PLOS ONE

Additional Editor Comments (optional):

Reviewers' comments:

# Editor report: Acceptance letter

**DOI:** 10.1371/journal.pone.0295966.r006

**Authors:** Chandrabose Selvaraj

29 Jan 2024

PONE-D-23-20740R2 

PLOS ONE

Dear Dr. Faya Castillo, 

I'm pleased to inform you that your manuscript has been deemed suitable for publication in PLOS ONE. Congratulations! Your manuscript is now being handed over to our production team.

At this stage, our production department will prepare your paper for publication. This includes ensuring the following:

* All references, tables, and figures are properly cited

* All relevant supporting information is included in the manuscript submission,

* There are no issues that prevent the paper from being properly typeset

If revisions are needed, the production department will contact you directly to resolve them. If no revisions are needed, you will receive an email when the publication date has been set. At this time, we do not offer pre-publication proofs to authors during production of the accepted work. Please keep in mind that we are working through a large volume of accepted articles, so please give us a few weeks to review your paper and let you know the next and final steps. 

Lastly, if your institution or institutions have a press office, please let them know about your upcoming paper now to help maximize its impact. If they'll be preparing press materials, please inform our press team within the next 48 hours. Your manuscript will remain under strict press embargo until 2 pm Eastern Time on the date of publication. For more information, please contact onepress@plos.org.

If we can help with anything else, please email us at customercare@plos.org.

Thank you for submitting your work to PLOS ONE and supporting open access. 

Kind regards, 

PLOS ONE Editorial Office Staff

on behalf of

Dr. Chandrabose Selvaraj 

Academic Editor

PLOS ONE
